# Supplementary material for: Impact of constitutional TET2 haploinsufficiency on molecular and clinical phenotype in humans
Source: Nat Commun. 2019 Mar 19;10:1252. doi: 10.1038/s41467-019-09198-7 (PMC6424975; doi:10.1038/s41467-019-09198-7)
Supplement: Supplementary file 1 — Supplementary Information [file 41467_2019_9198_MOESM1_ESM.pdf]

Supplementary Information

Impact of Constitutional *TET2* Haploinsufficiency on Molecular and Clinical Phenotype in Humans

Kaasinen, Kuismin, Rajamäki *et al.*

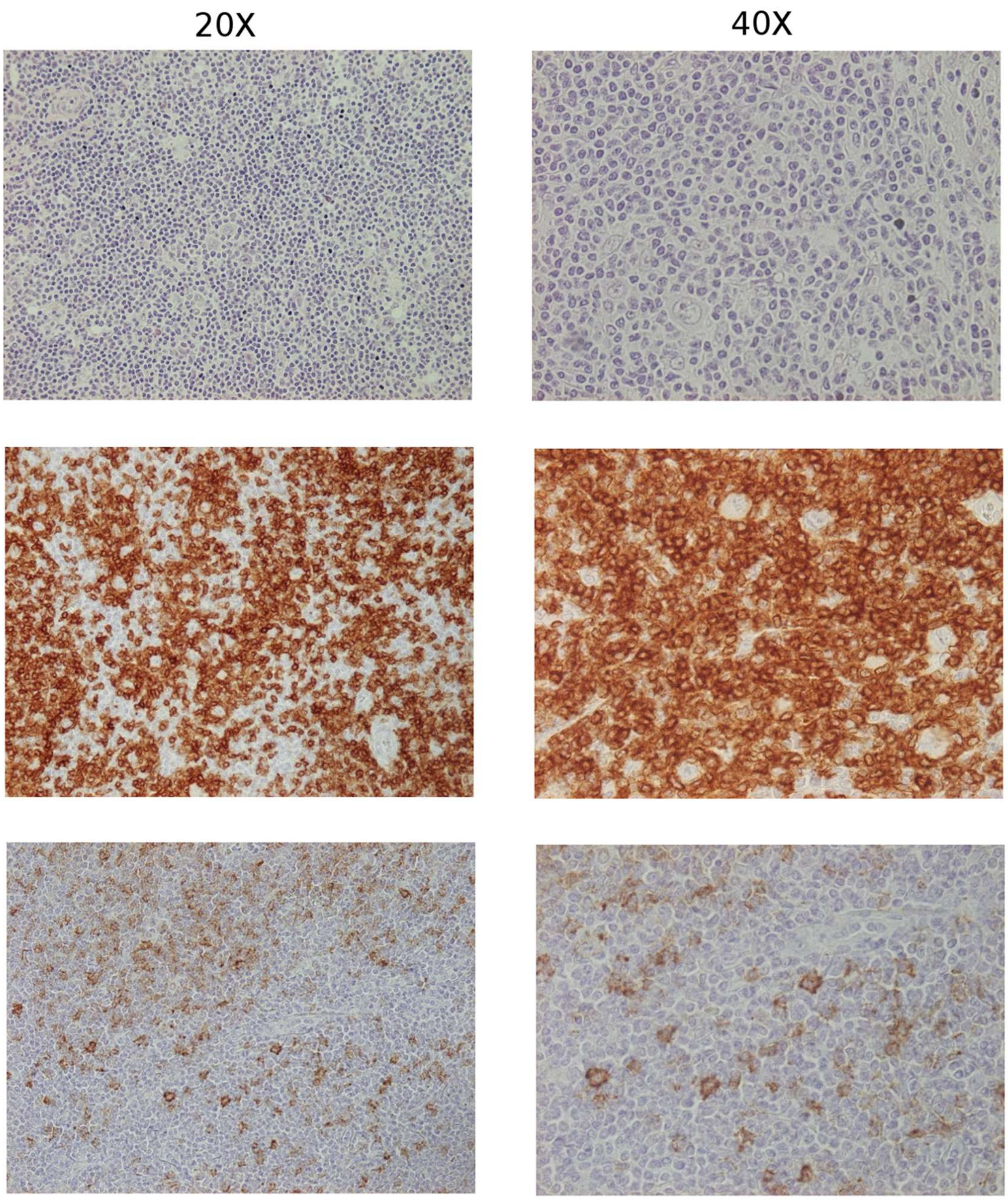

Supplementary Figure 1. Immunohistochemical stainings on tumor biopsies from nodular lymphocyte predominant Hodgkin lymphoma (NLPHL) patient Ly1. Hematoxylin and eosin (HE, upper panel), CD3 (middle panel), and CD20 (lower panel) stained sections from NLPHL tumor biopsies. The malignant lymphocyte predominant (LP) cells are large atypical cells with popcorn kernel-like structure. They comprise only a small fraction of cells in tumor tissue. The LP cells are often surrounded by CD3-positive lymphocytes forming rosettes around them. The LP cells also stain positively with CD20, but also most of the surrounding lymphocytes are positive for CD20. The images were taken with Leica DM LB microscope using 20x (left column) and 40x (right column) magnification.

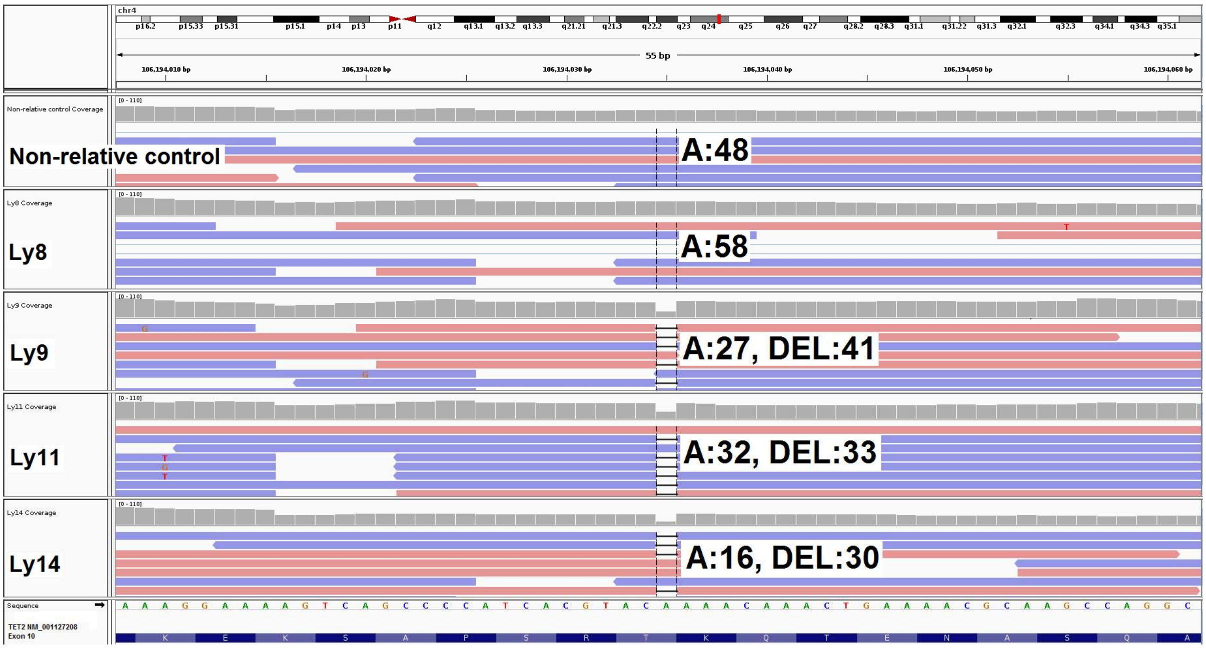

Supplementary Figure 2. Expression of *TET2* deletion in macrophages. Mutation carriers (Ly9, Ly11 and Ly14) have similar or slightly higher expression of the mutant allele than the wild-type allele. Note that both alleles are present due to escape from nonsense mediated mRNA decay as expected based on the location of the stop codon. Blue reads align to bottom strand and red reads align to top strand at the *TET2* deletion locus of human genome hg19.

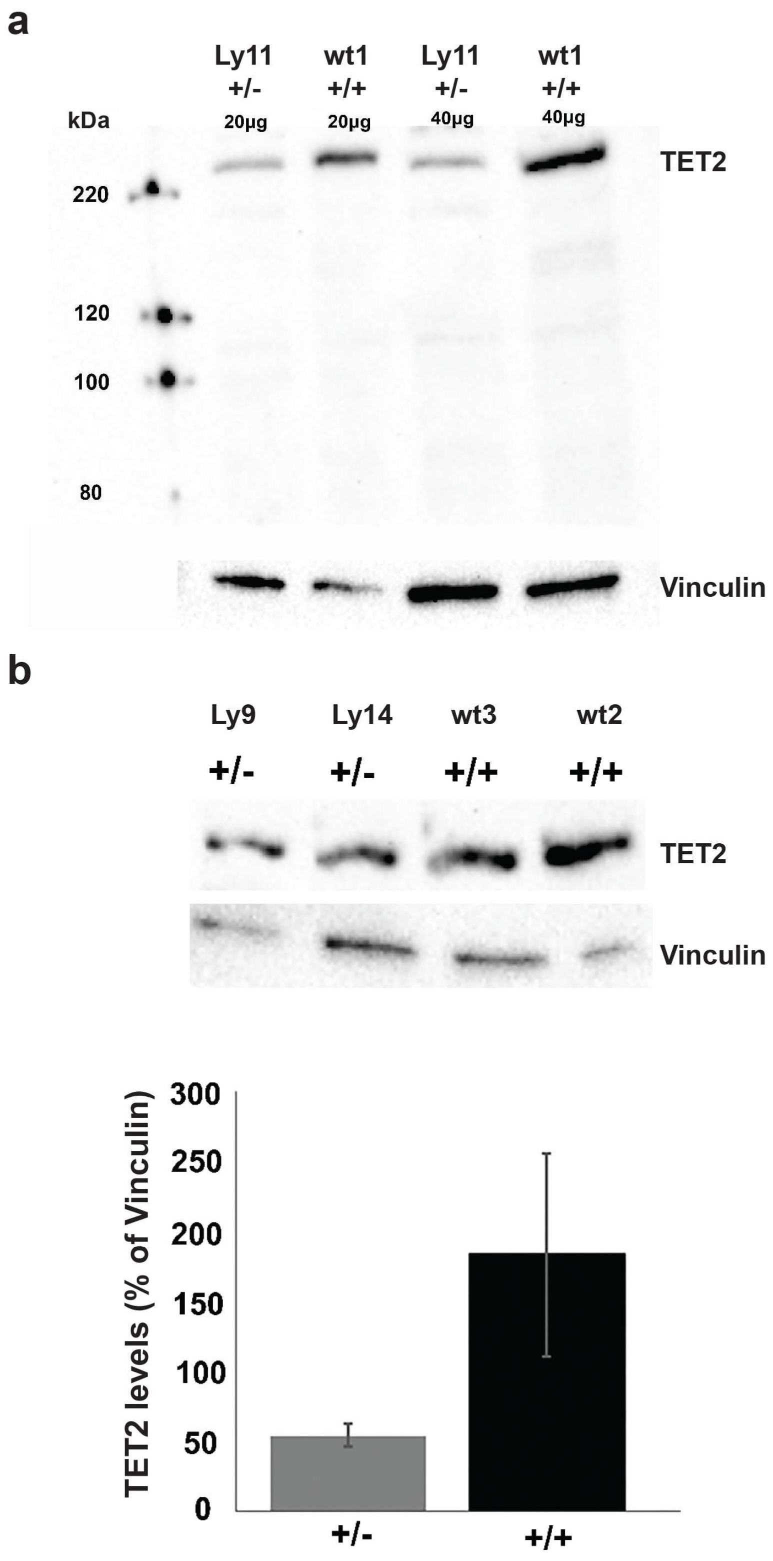

Supplementary Figure 3. TET2 western blot. (**a**) Full gel image of extended TET2 western blot run. Total protein extracts (20 and 40 µg) are from lymphoblastoid cells of one mutation carrier (+/-) and one wild-type (+/+) individual of the family. Only the wild-type form of TET2 protein at 260 kDa is identified. Vinculin was used as a loading control. (**b**) Replicate western blot of TET2 from lymphoblastoid cells of two TET2delA mutation carriers and two wild-type individuals included also in **Fig. 1g**. The graph shows the quantification of TET2 protein normalized to the vinculin. Bars represent the mean +/- s.d. of TET2 intensities.

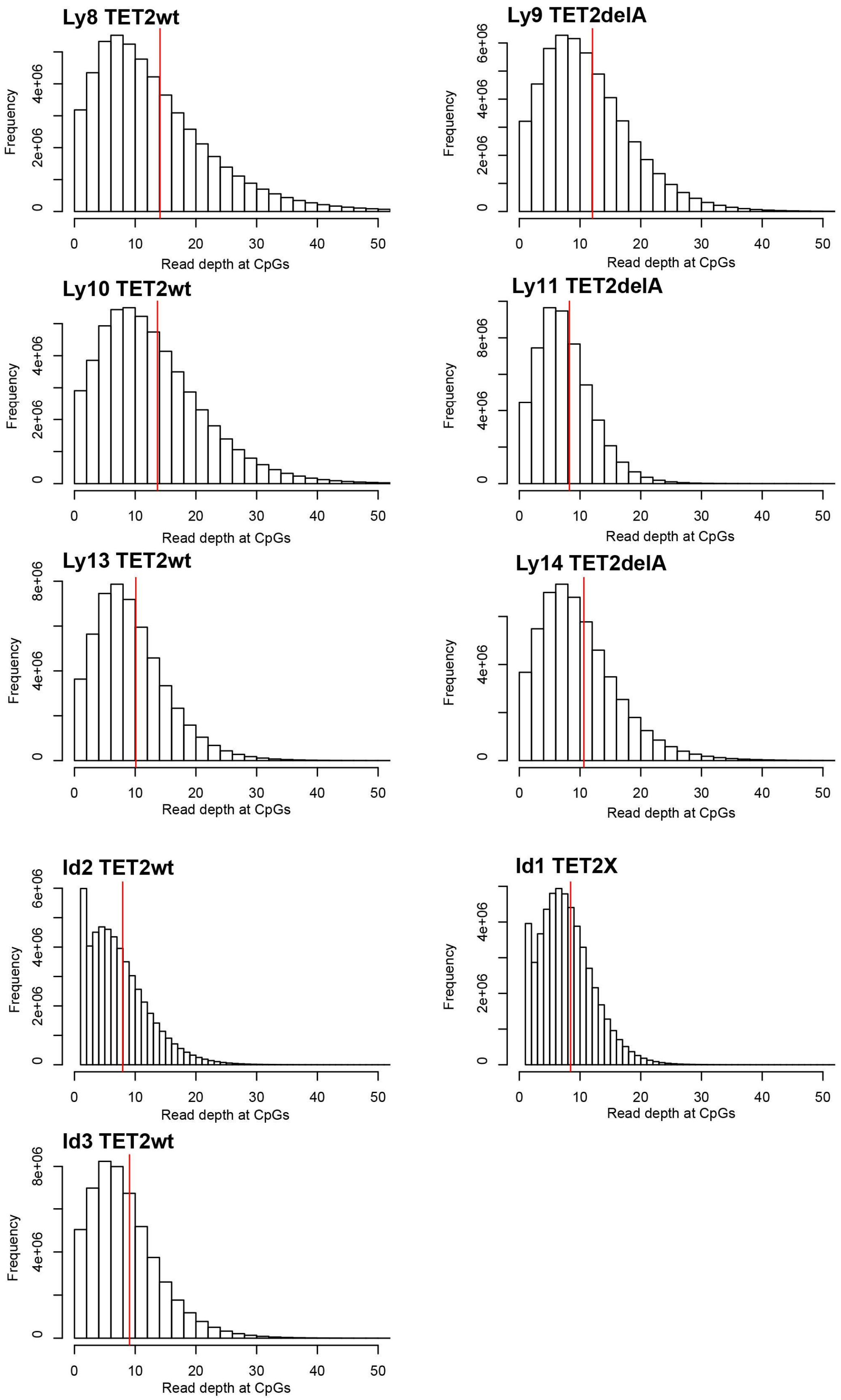

Supplementary Figure 4. Histograms of depth of coverage of non-overlapping paired-end reads at CpGs from whole-genome bisulfite sequenced samples. Red line indicates average coverage.

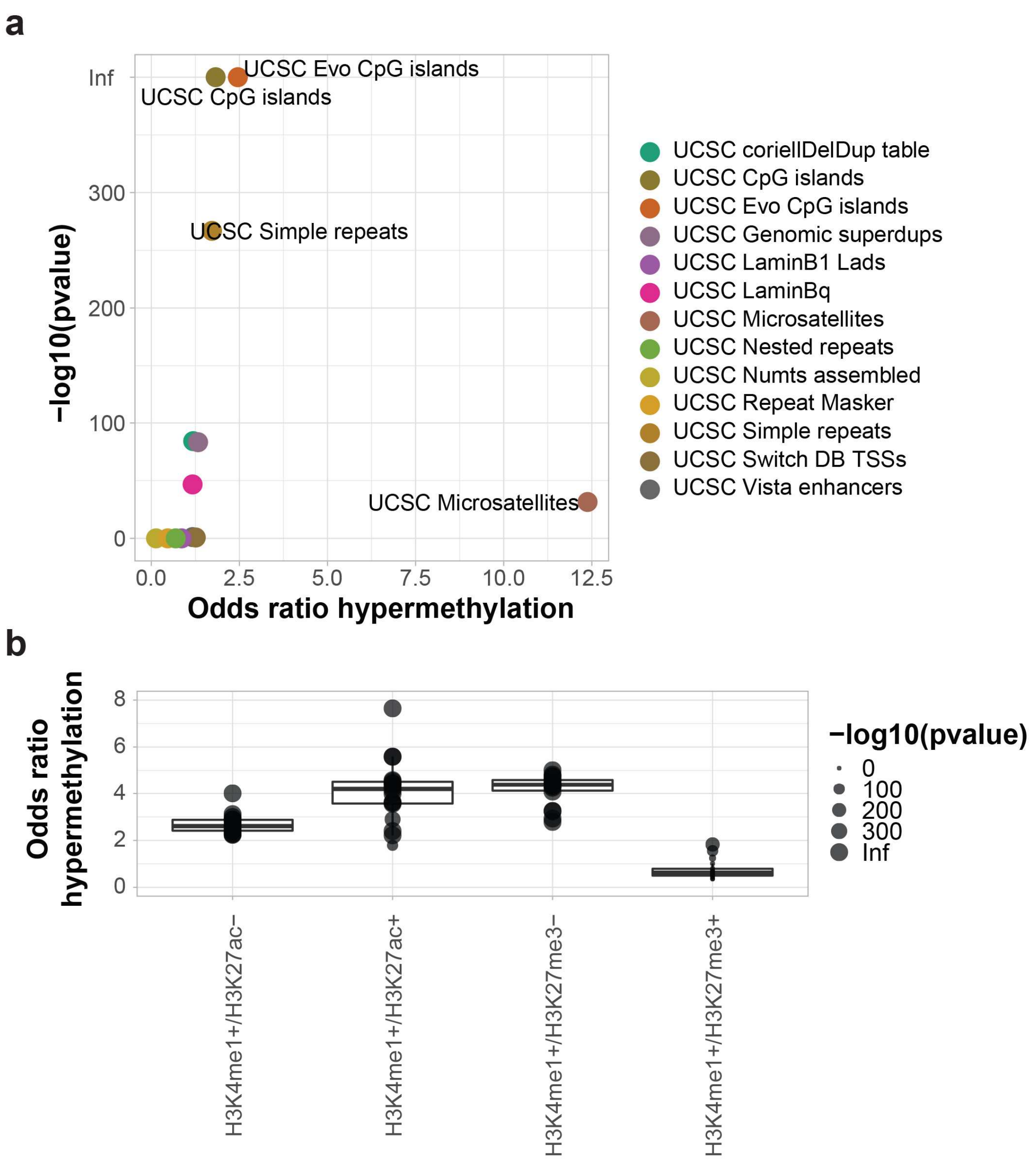

Supplementary Figure 5. Enrichment of hypermethylation associated with TET2 loss at different genomic annotations.

Hypermethylated CpGs were derived from comparison of three TET2delA carriers to three age-matched non-carriers included in whole-genome bisulfite sequencing. Odds ratios and p-values are calculated with the Fisher's exact test implemented in LOLA R package. (**a**) Hypermethylation shows enrichment at CpG islands and to some degree at simple repeats. Annotations of the hg19 genome were derived from UCSC Genome Browser. (**b**) Hypermethylation shows enrichment at H3K4me1 regions without H3K27me3 in primary blood cells. Regions with different chromatin marks in 24 different subtypes of primary human blood cells (each subtype represented by a dot) were available from the Roadmap Epigenomics project. Boxplots show the median, and the first and third quartiles.

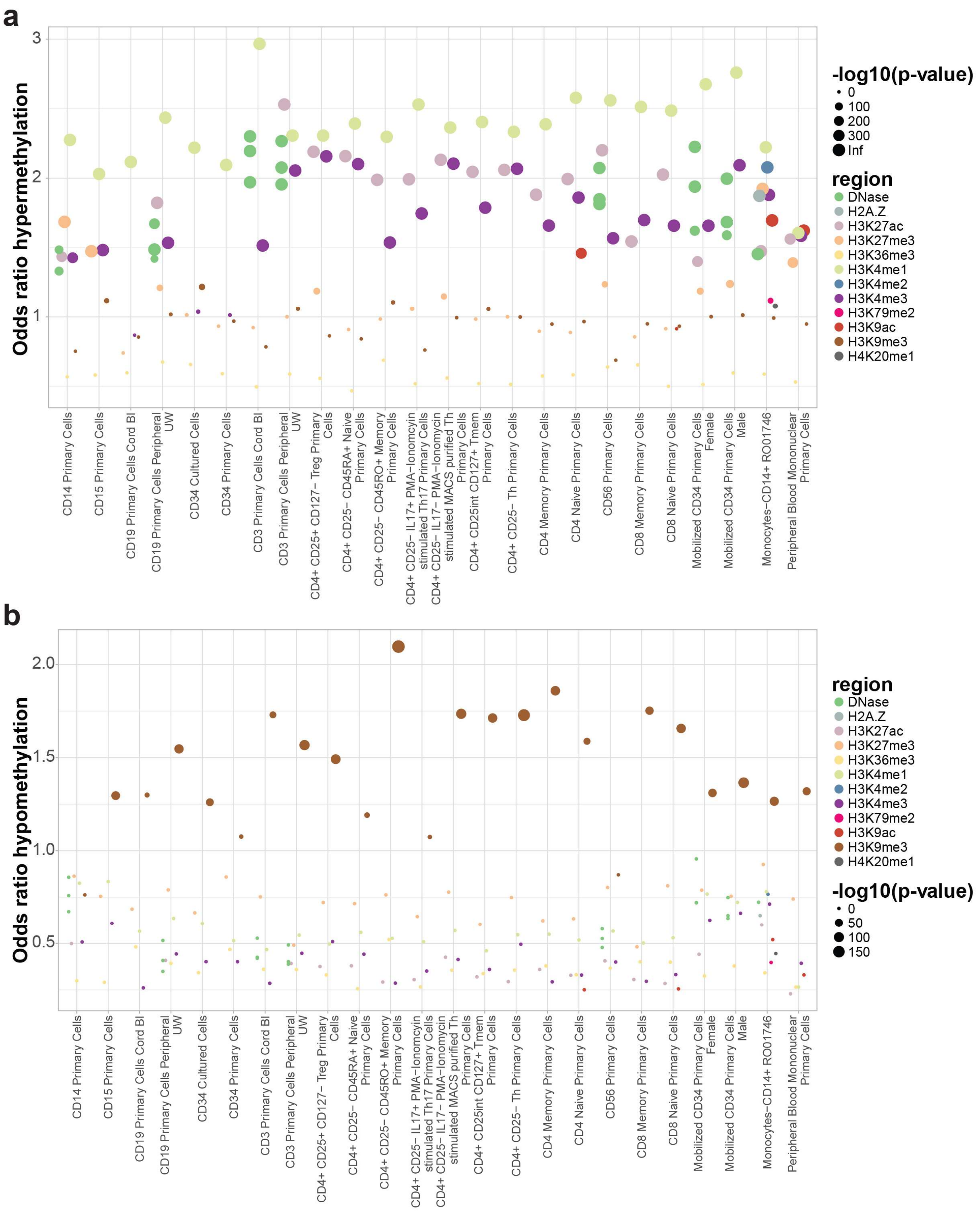

Supplementary Figure 6. Enrichment of differentially methylated CpGs of Ly9 as compared to age-matched control at regions with different chromatin marks in primary blood cells. Enrichment of (**a**) hypermethylation and (**b**) hypomethylation as detected by LOLA R package.

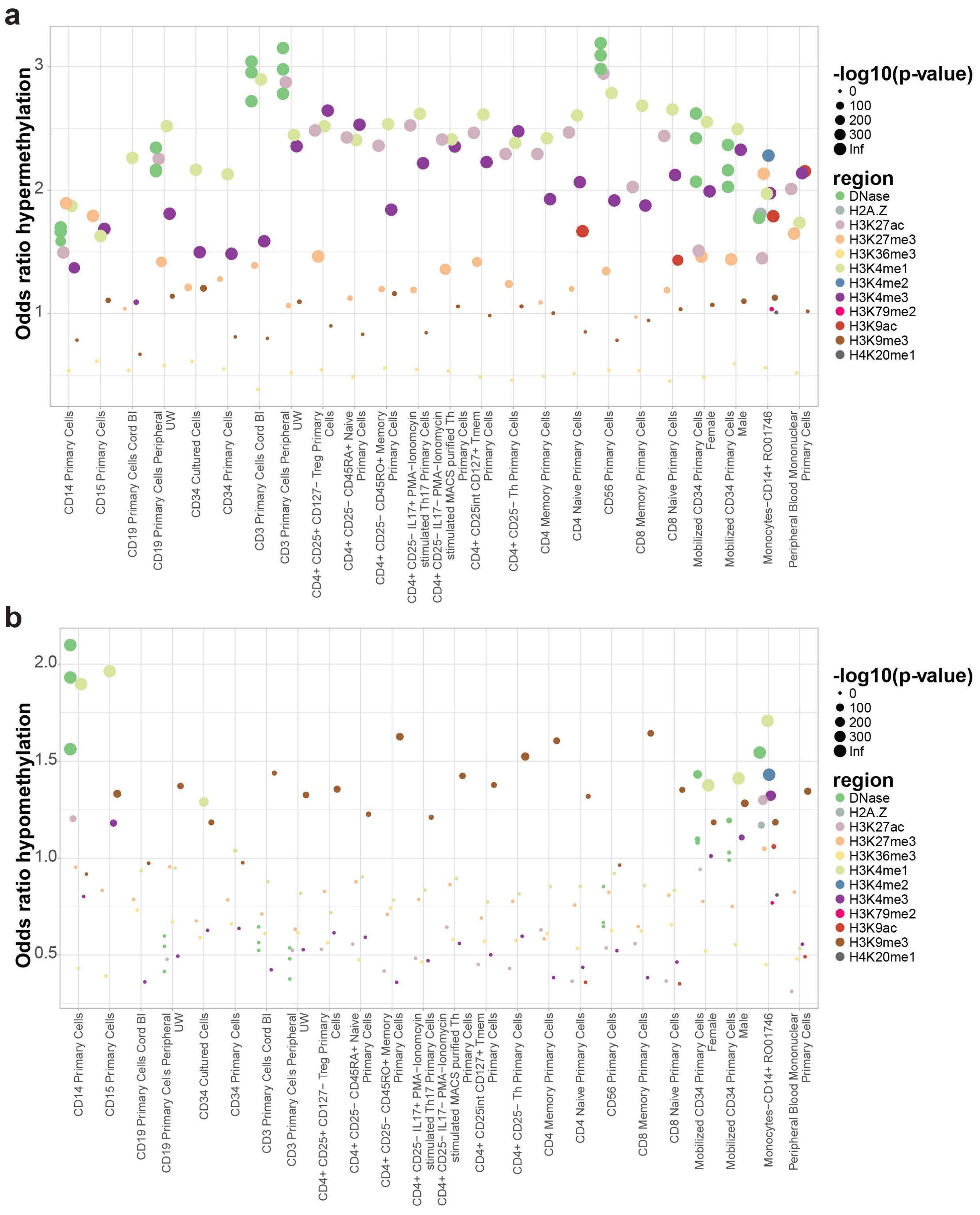

Supplementary Figure 7. Enrichment of differentially methylated CpGs of Ly11 as compared to age-matched control at regions with different chromatin marks in primary blood cells. Enrichment of (**a**) hypermethylation and (**b**) hypomethylation as detected by LOLA R package.

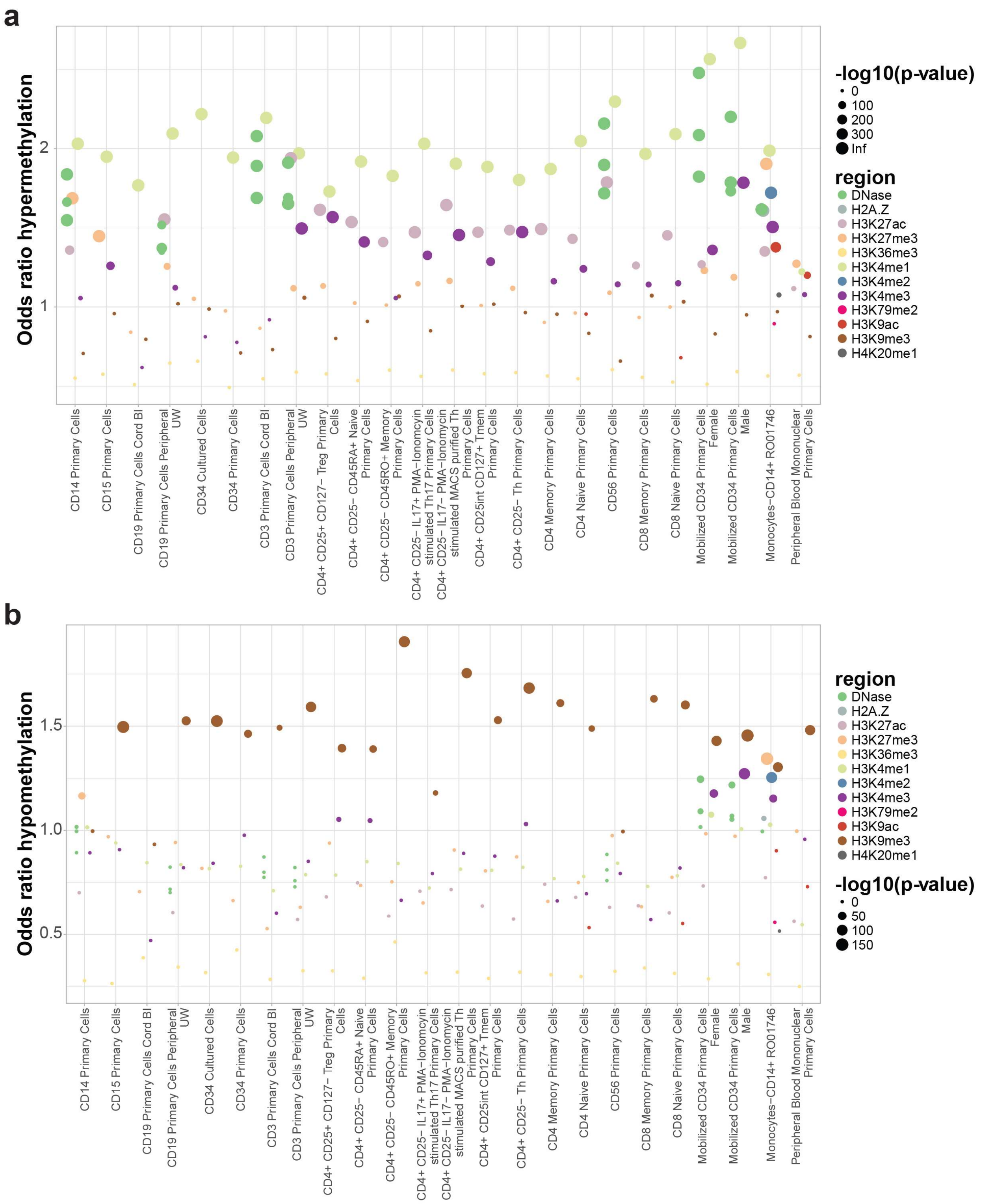

Supplementary Figure 8. Enrichment of differentially methylated CpGs of Ly14 as compared to age-matched control at regions with different chromatin marks in primary blood cells. Enrichment of (**a**) hypermethylation and (**b**) hypomethylation as detected by LOLA R package.

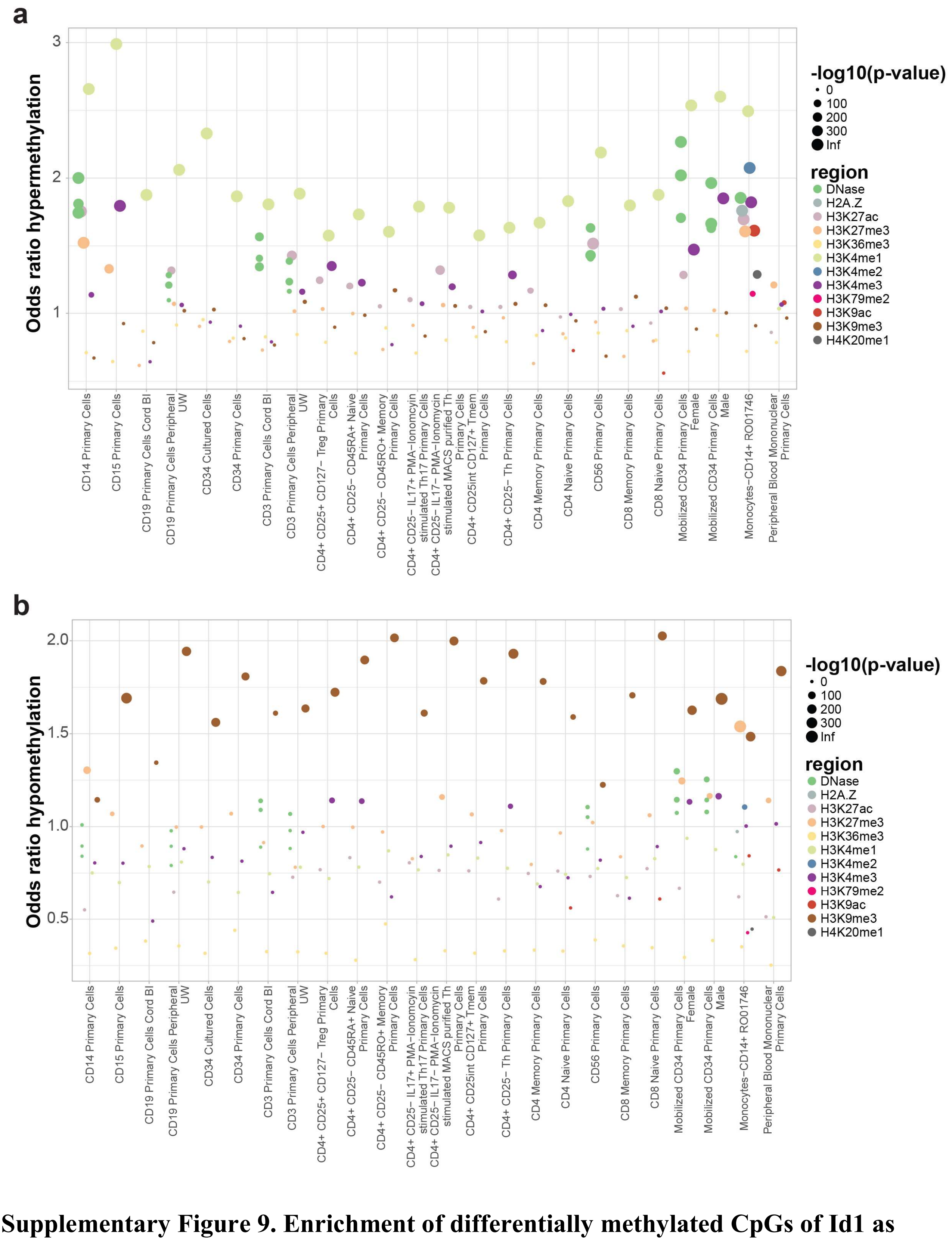

Supplementary Figure 9. Enrichment of differentially methylated CpGs of Id1 as compared to two age-matched controls at regions with different chromatin marks in primary blood cells. Enrichment of (**a**) hypermethylation and (**b**) hypomethylation as detected by LOLA R package.

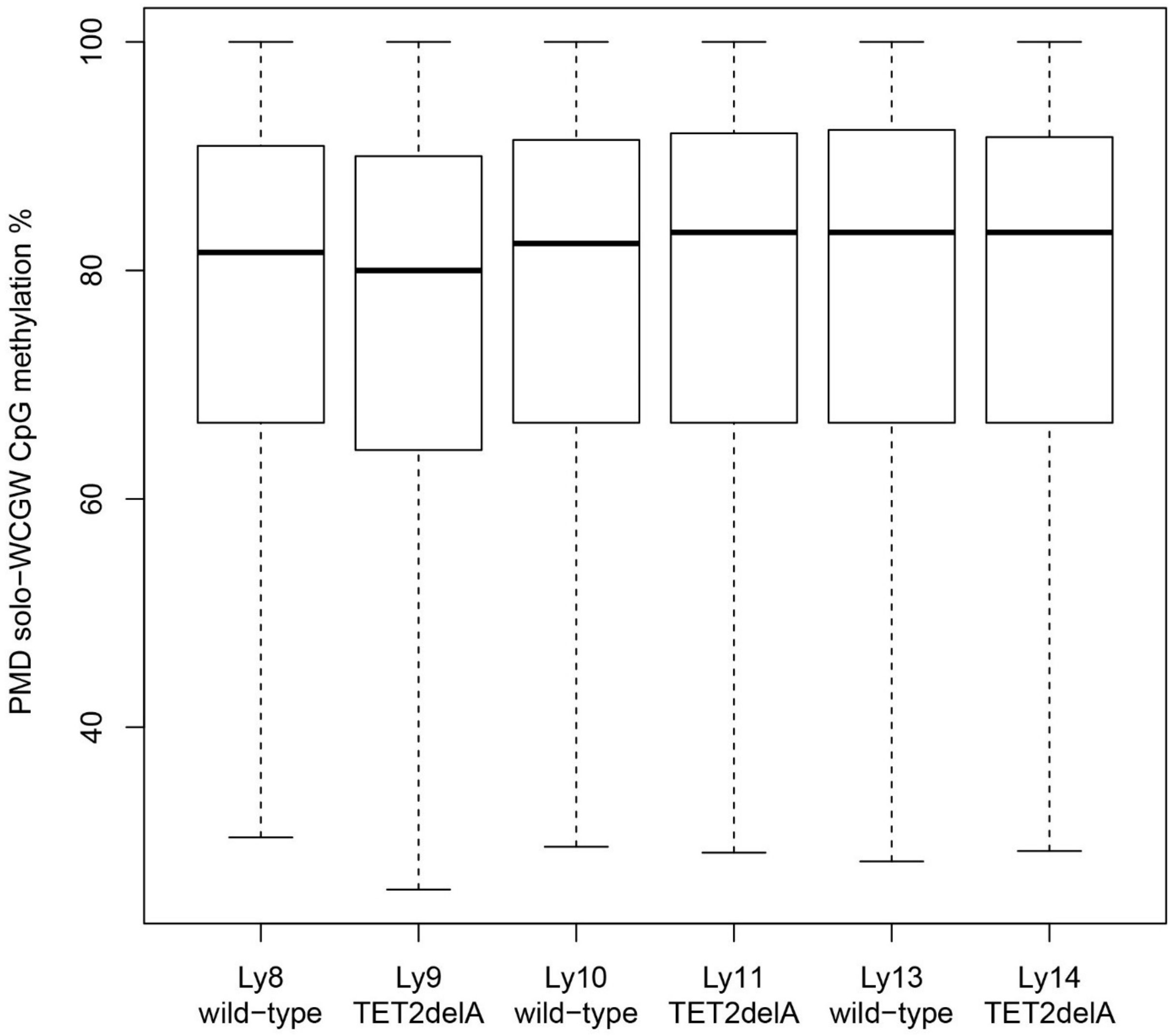

Supplementary Figure 10. Boxplot of methylation pergentages at CpGs associated with decreasing methylation in cellular aging.

Solo-WCGW CpGs represent sites with the combination of zero neighboring CpGs (‘solo’) and flanked by an A or T (‘W’) on both sides in common partially methylated domains (PMDs). Boxplots show the median, and the first and third quartiles.

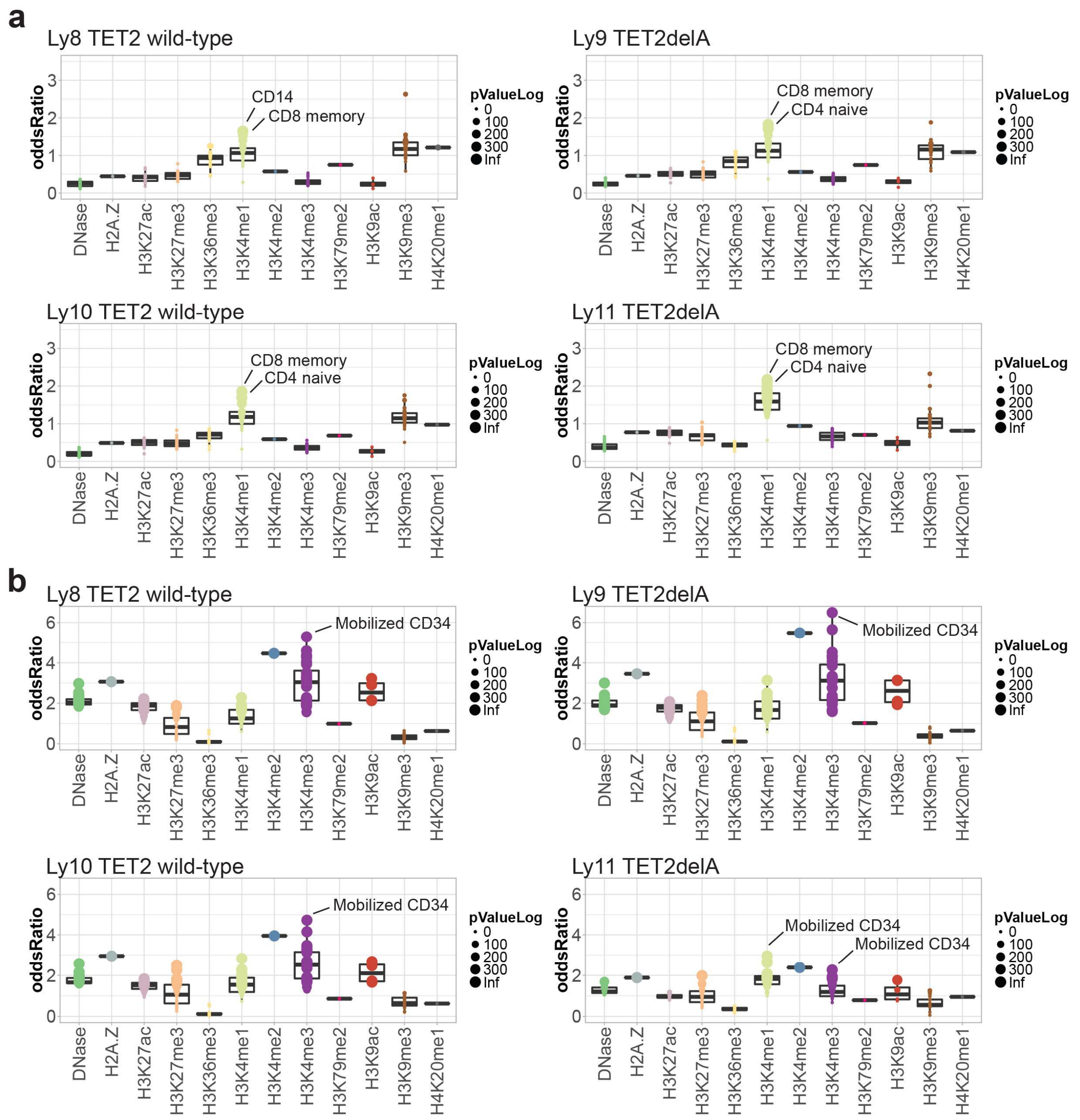

Supplementary Figure 11. Methylation changes during ten years' time interval at regions with different chromatin marks in primary blood cells. Enrichment of age-related (**a**) hypermethylation and (**b**) hypomethylation in each individual that was sampled twice at ten years' time interval. Cell types that show highest enrichment of age-related methylation changes at specific chromatin marks are indicated. Regions with different chromatin marks in 24 different subtypes of primary human blood cells (each subtype represented by a dot) were available from the Roadmap Epigenomics project. Odds ratios and p-values from the Fisher's exact test implemented in LOLA R package.

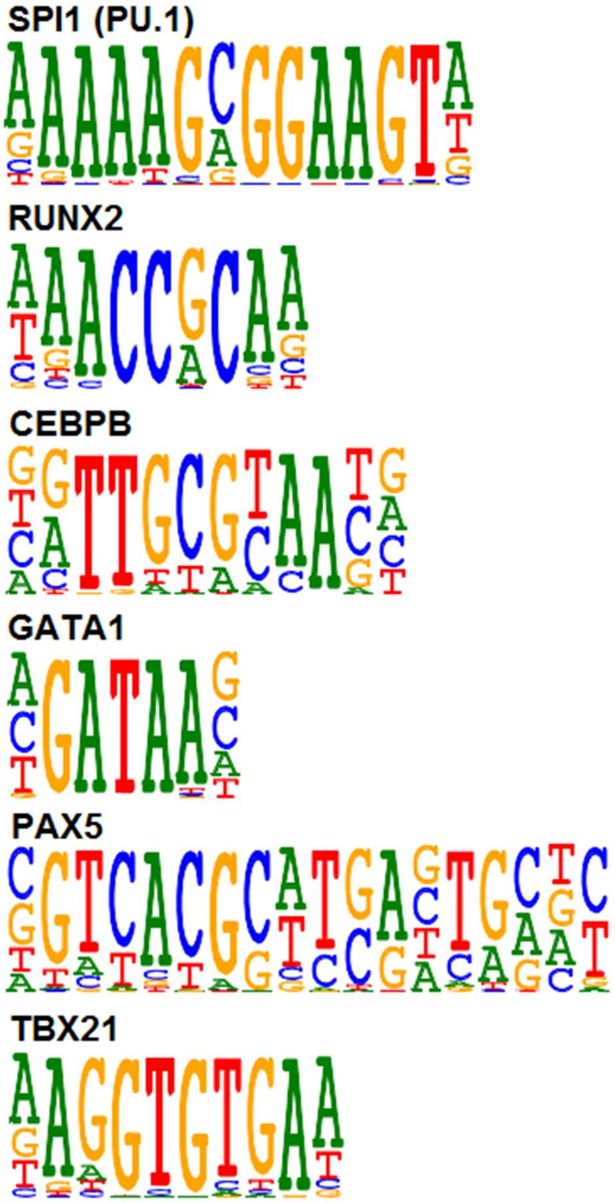

Supplementary Figure 12. Sequence logos of SPI1 (PU.1), RUNX2, CEBPB, GATA1, PAX5 and TBX21 binding sites used in motif mapping.

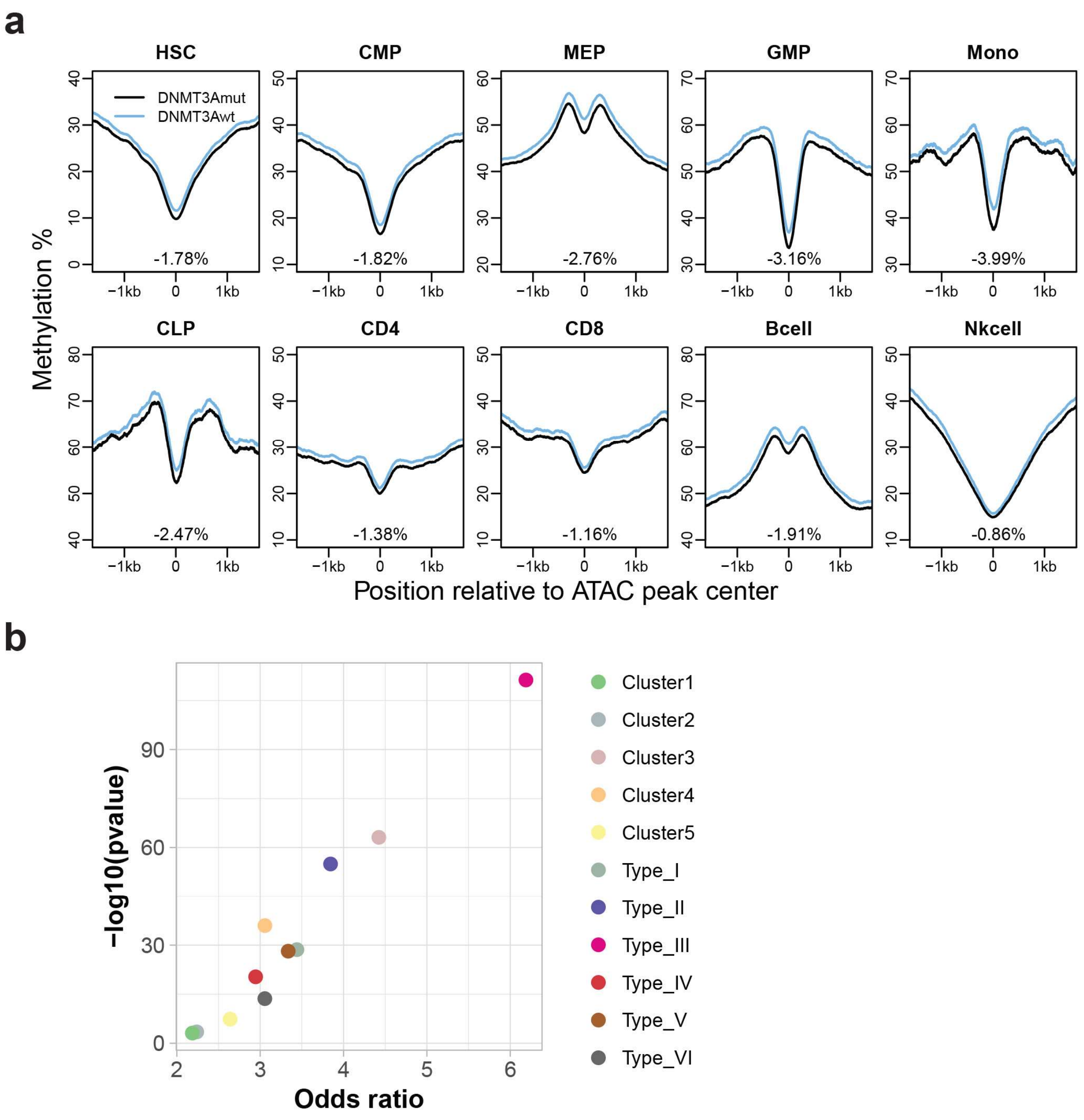

Supplementary Figure 13. DNMT3A mediated methylation changes per cell lineage and relation of DNMT3A and TET2 actions. (**a**) Methylation is decreased in *DNMT3A* mutation carriers at open chromatin regions of all blood cell types, and in particular, monocytes. Percentages represent average methylation differences at the respective open chromatin regions between four *DNMT3A* mutation carriers (Id5, Id7, Id9, Id11) and four controls from NFID cohort (Id6, Id8, Id10, Id12). CLP: common lymphoid progenitor; CMP: common myeloid progenitor; GMP: granulocyte/macrophage progenitor; HSC: hematopoietic stem cell; MEP: megakaryocyte/erythroid progenitor; Mono: monocyte. (**b**) Hypermethylation derived from whole-genome bisulfite sequencing of three TET2delA carriers and three age-matched non-carriers is enriched at regions with synergistic (Type_III in Zhang et al.[1]) and competitive (Cluster3 in Zhang et al.[1]) action of DNMT3A and TET2. Odds ratios and p-values from the Fisher's exact test implemented in LOLA R package.

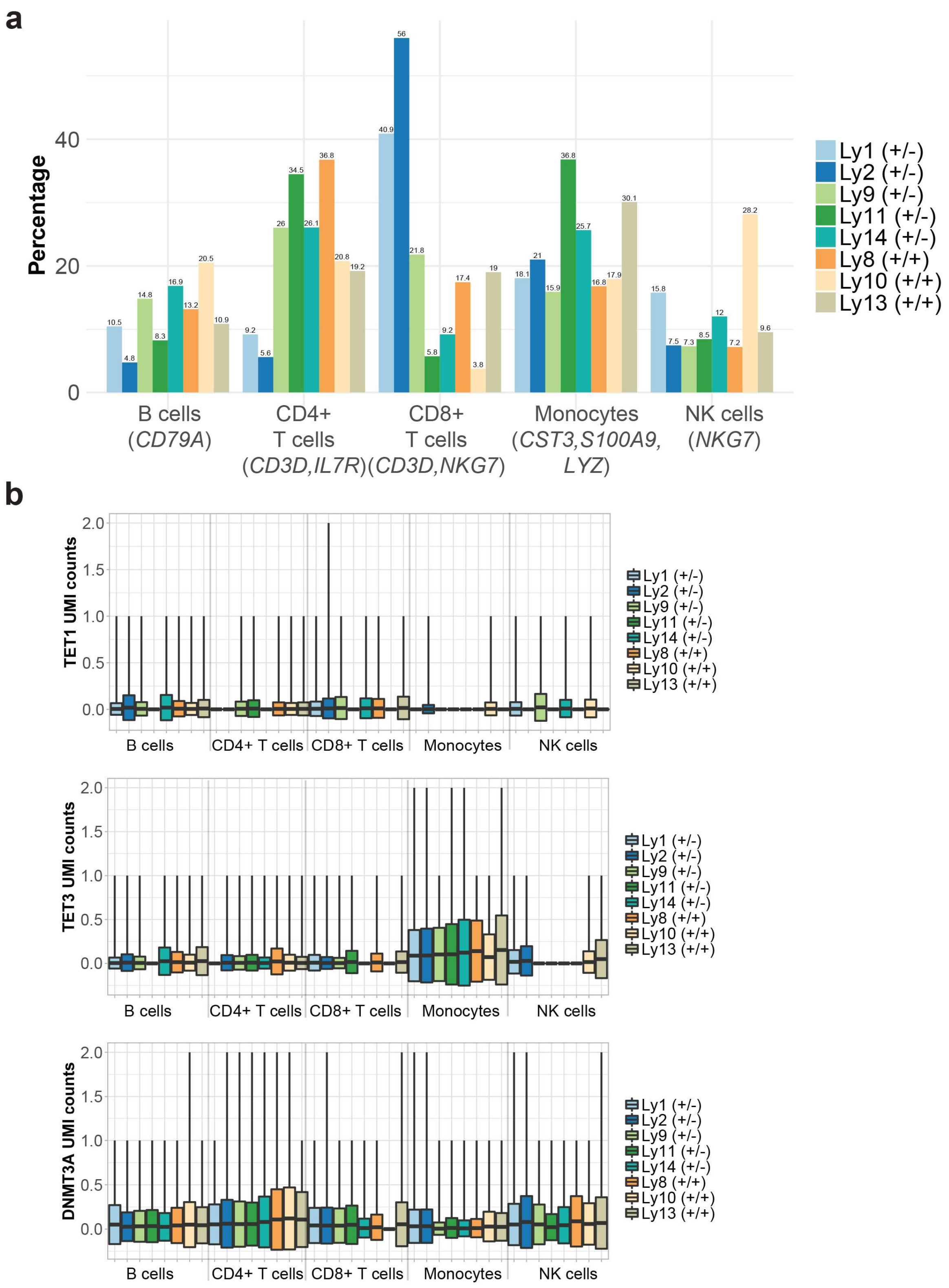

Supplementary Figure 14. Single-cell RNA-sequencing analysis of peripheral blood cells. Major blood cell types were identified utilizing cluster-specific gene expression patterns. Cells from all individuals were clustered with K-mean (K=10) clustering. (**a**) The fractions of the major blood cell types do not show consistent differences between *TET2* c.4500delA mutation carriers (+/-) and wild-type individuals (+/+). (**b**) *TET1*, *TET3* and *DNMT3A* expression per individual in each of the identified cell types. *DNMT3A* displays

slight expression decrease in the mutation carriers as compared to wild-type individuals. Boxplots show the mean +/- standard deviation.

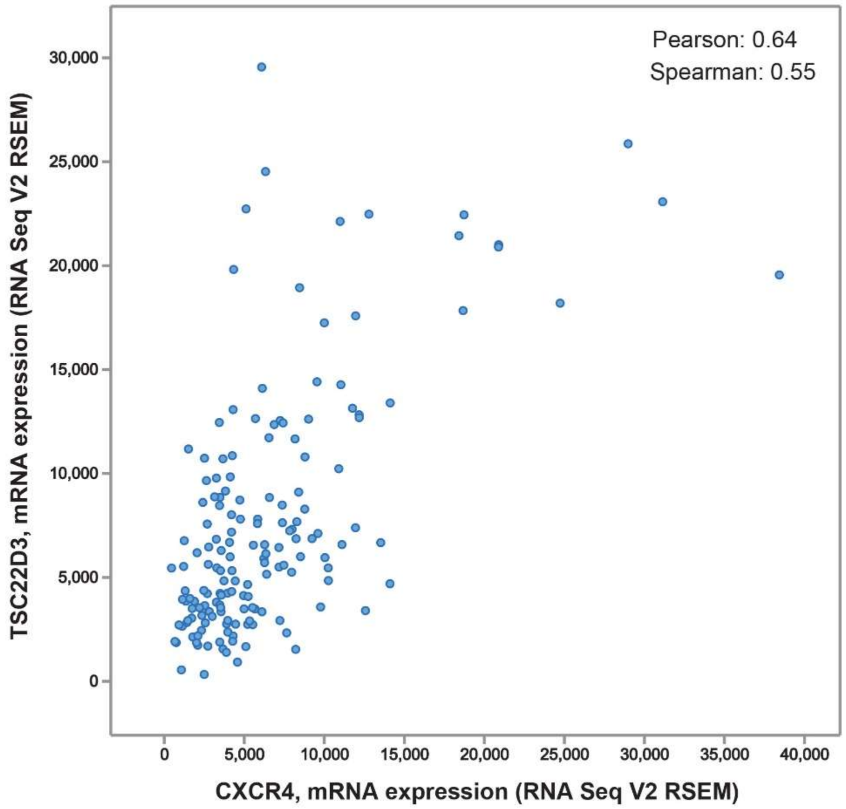

Supplementary Figure 15. Correlation of expression of *CXCR4* and *TSC22D3* in acute myeloid leukemia.

The most correlated gene with *CXCR4* is *TSC22D3* in the RNA-sequencing data of 162 acute myeloid leukemia samples[2].

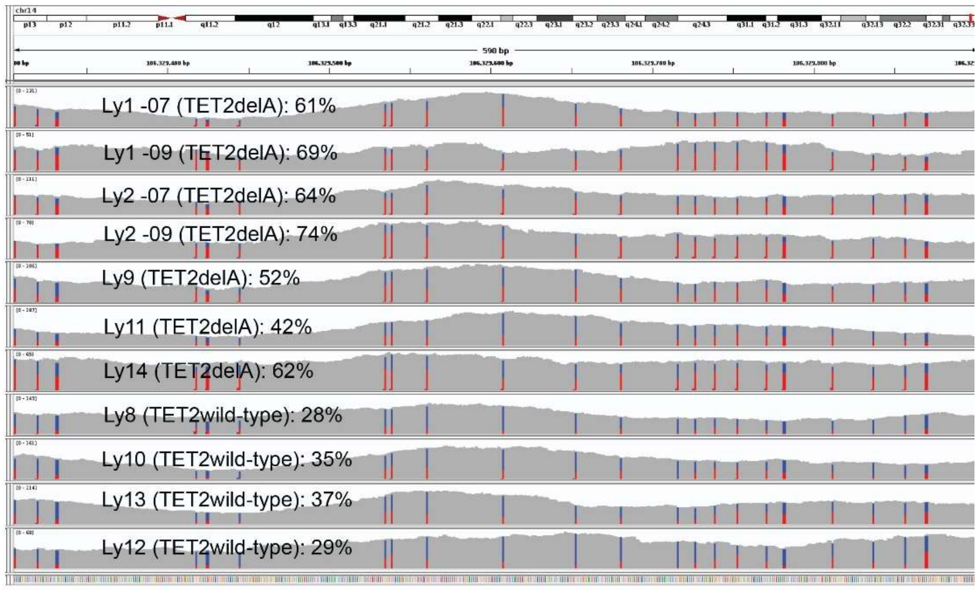

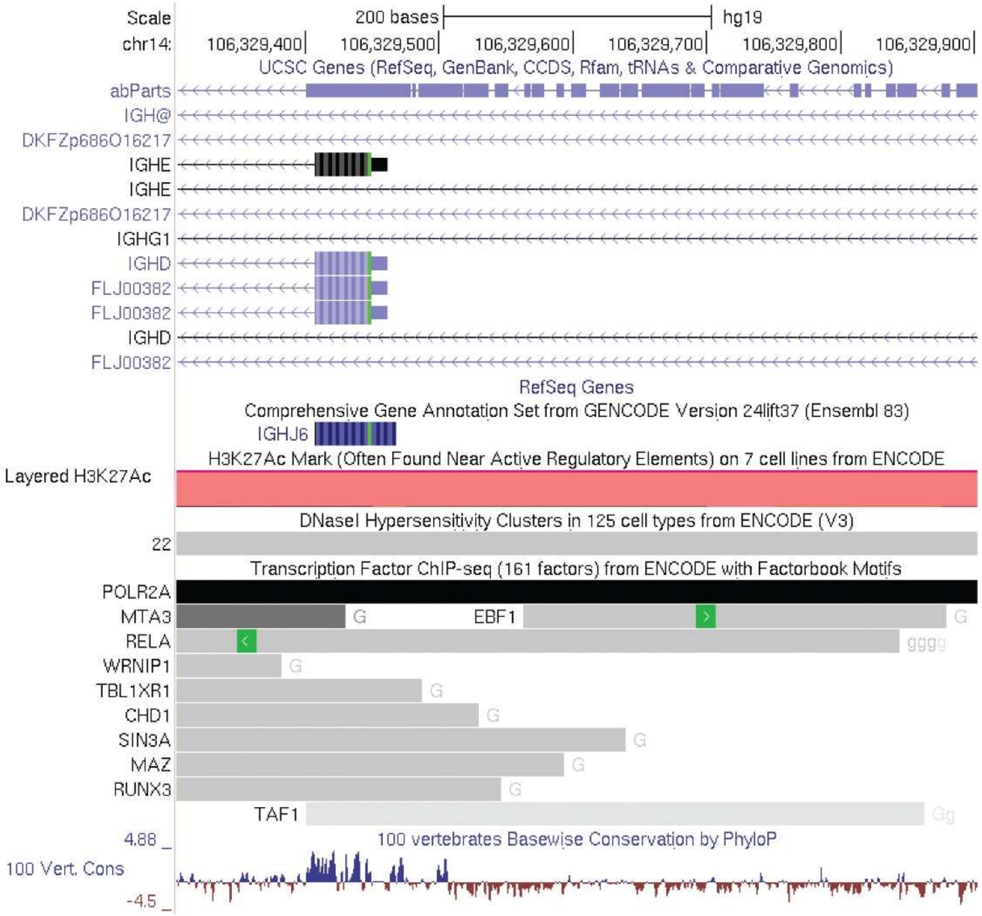

Supplementary Figure 16. Differentially methylated region at IGH locus. (**a**) Percentages of methylated CpGs within significantly differentially methylated region at the promoter of *IGHJ6*. Average methylation percentages are shown with sample identifiers. Red bars represent methylated C and blue bars represent unmethylated C at each CpG in aligned bisulfite sequenced reads (grey). (**b**) UCSC Genome Browser view of the differentially methylated region reveals promoter characteristics. H3K27Ac, POLR2A and TAF1 signals originate from ENCODE GM12878 lymphoblastoid cell line.

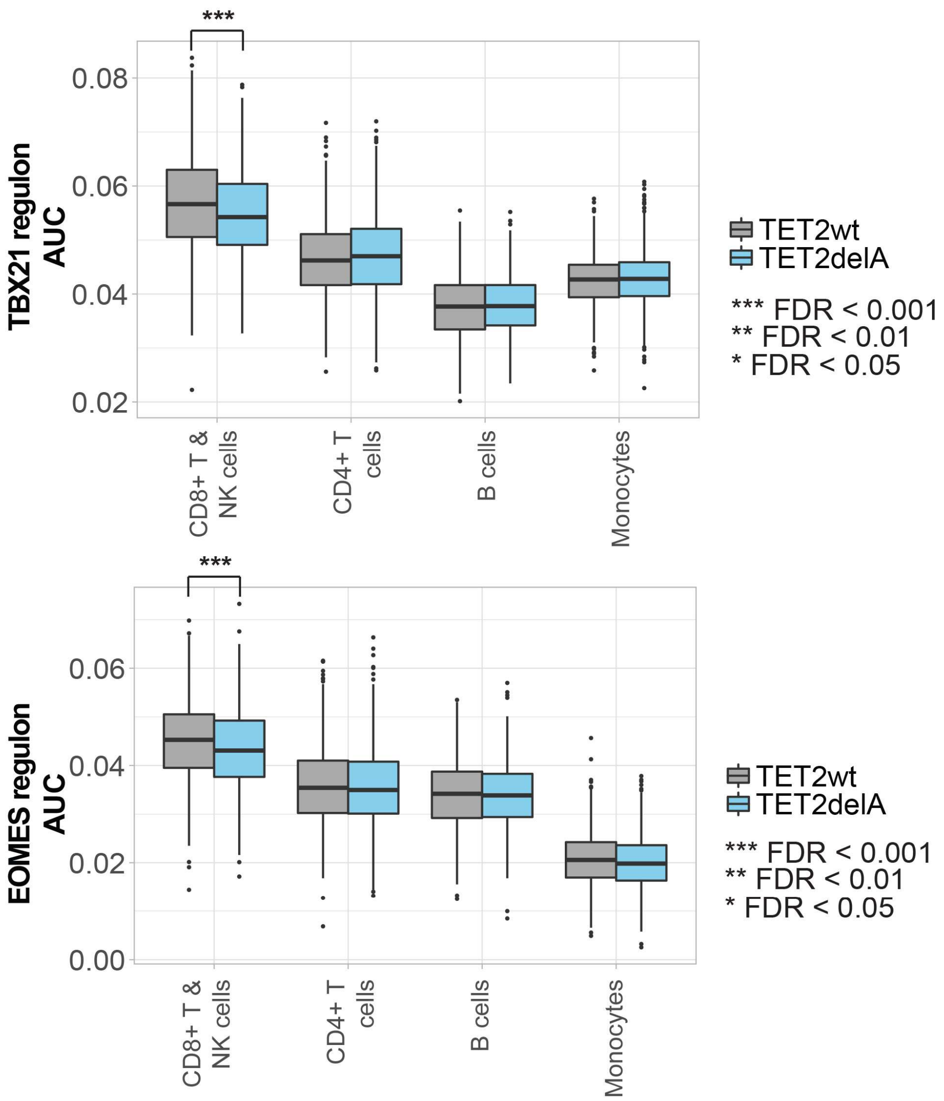

Supplementary Figure 17. Regulon activity of TBX21 and EOMES. Significance of regulon activity difference in each cell type is calculated with Wilcoxon rank sum test between three cancer-free TET2delA carriers and three age-matched wild-type (wt) individuals. Boxplots show the median, and the first and third quartiles.

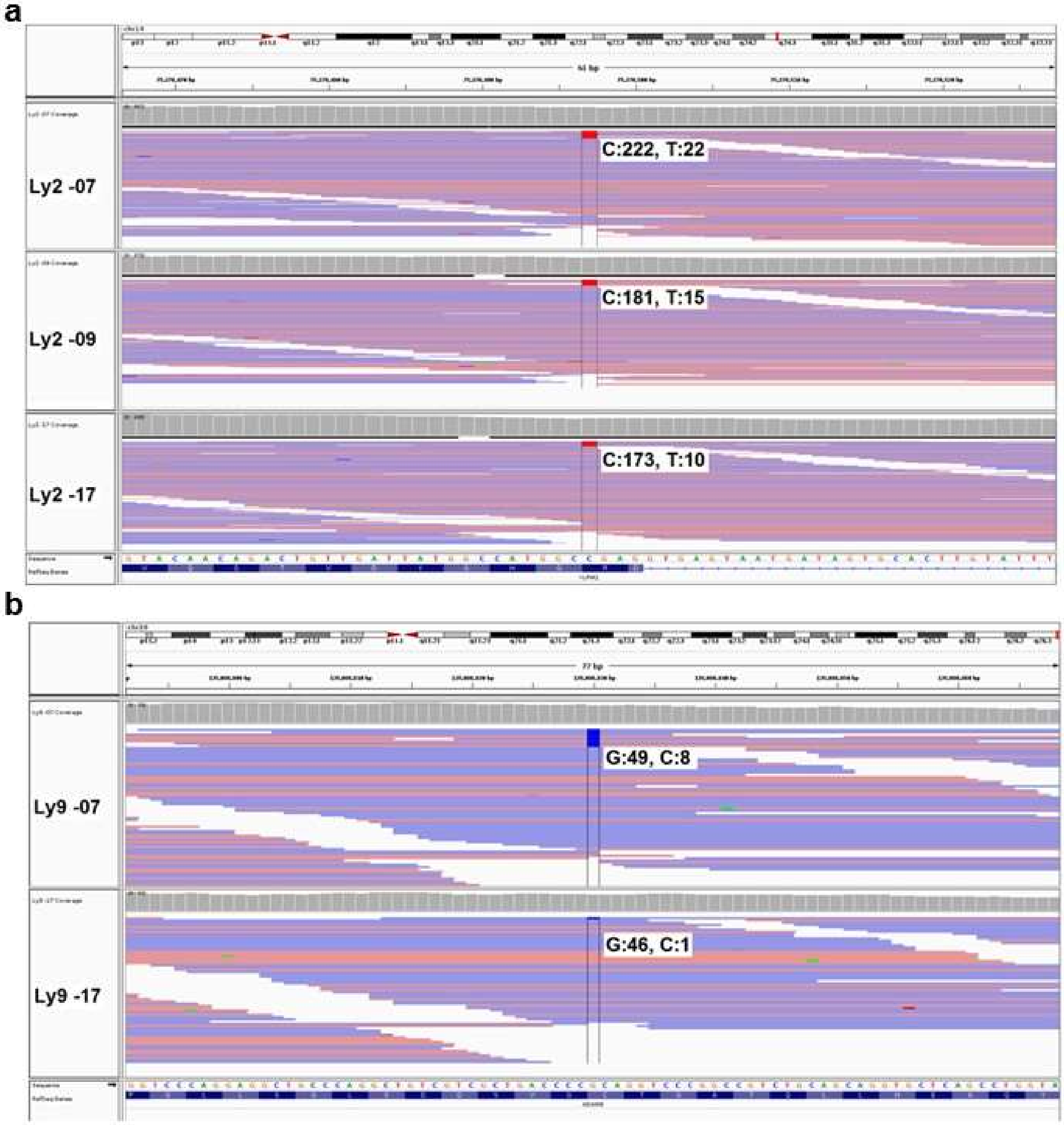

Supplementary Figure 18. Variants indicating clonal hematopoiesis. (**a**) Subclonal nonsense mutation p.Arg1646Ter in *YLPM1* was detected in Ly2 by deep exome sequencing of blood DNA extracted in years 2007, 2009 and 2017. (**b**) Subclonal missense mutation p.Cys167Trp in *ADAM8* was detected in Ly9 by deep exome sequencing of blood DNA extracted in years 2007 and 2017. The missense mutation was predicted to be damaging by Provean and SIFT. The mutations were not detectable in the deep sequencing data from other family members or in-house control data. Blue reads align to bottom strand and red reads align to top strand at the respective loci of human genome hg19.

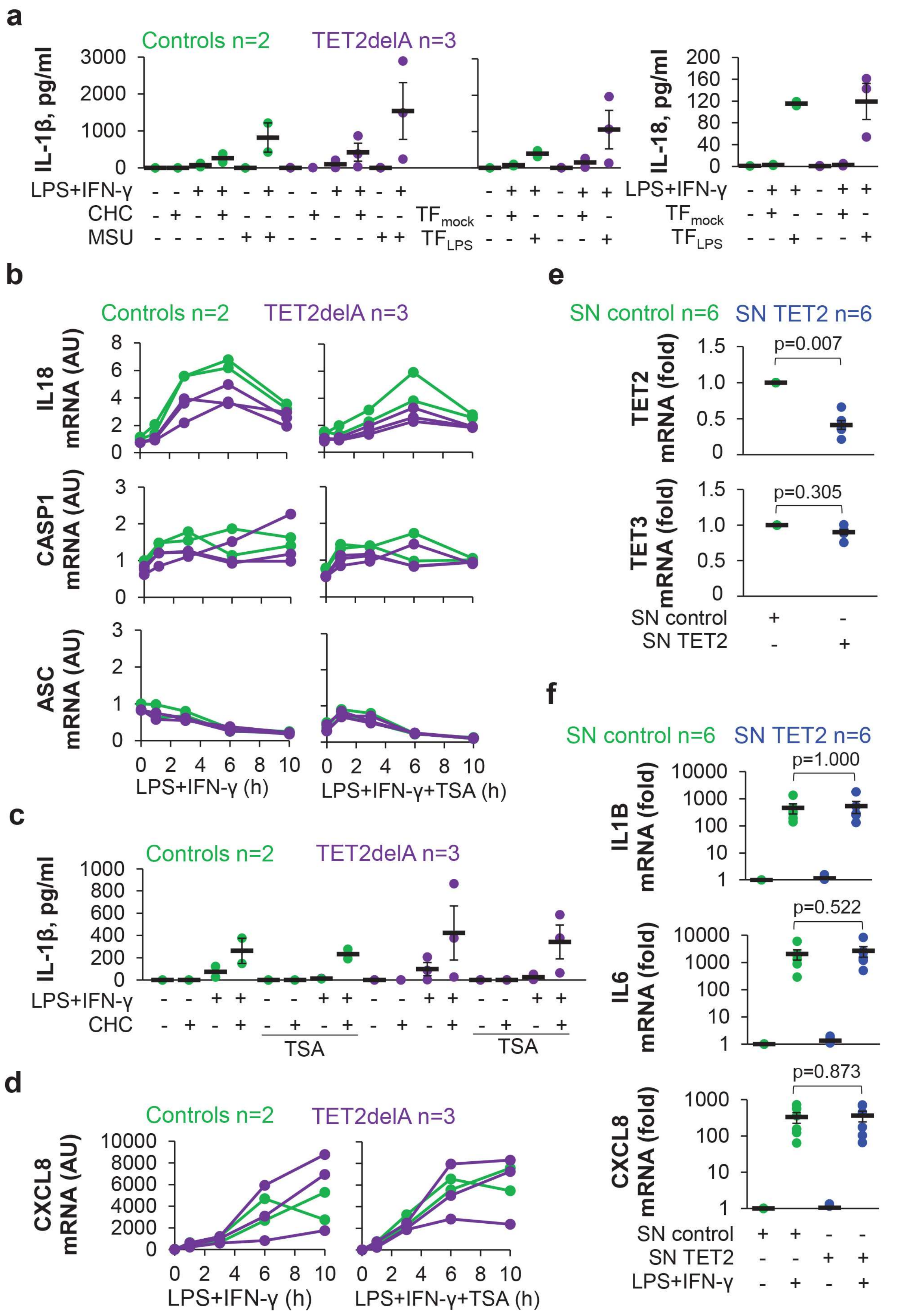

Supplementary Figure 19. Macrophage inflammasome-mediated cytokine response is not elevated in constitutional heterozygous *TET2* loss. Cytokine

responses in monocyte-derived macrophages of (**a-d**) TET2delA mutation carriers and controls and (**e, f**) normal human donors transfected with control or TET2 siRNAs. (**a, c**) Macrophages were primed for 6 h with lipopolysaccharides (LPS) and interferon-γ (IFNγ) followed by NLRP3 inflammasome activation with cholesterol crystals (CHC), monosodium urate crystals (MSU), or by transfection (TF) of LPS to cytoplasm. Secretion of mature IL-1β and IL-18 was measured by ELISA from cell culture supernatants. (**b, d-f**) Macrophages were left untreated or treated for the indicated times with LPS and IFN-γ in the presence or absence of trichostatin A (TSA), and mRNA expression was analyzed by qPCR (SN, siRNA; AU, arbitrary units; fold, fold change to cells transfected with control siRNA). Individual data points from each donor are shown with mean values +/- s.e.m. (**e, f**) Statistical analysis was performed with Wilcoxon rank sum test.

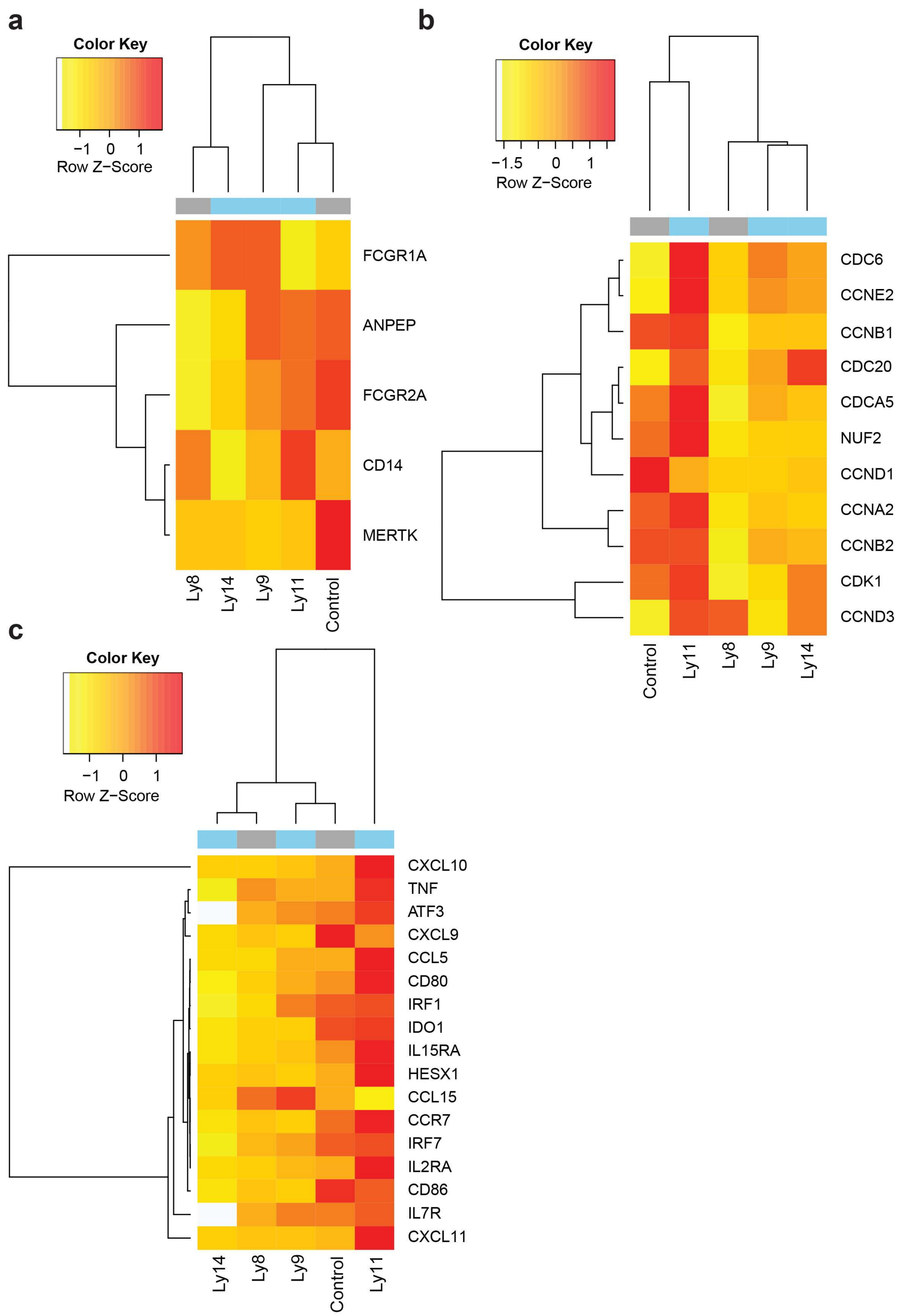

Supplementary Figure 20. Additional analyses in macrophages from *TET2* mutation carriers and controls. Expression heatmap of gene sets representing macrophage differentiation (**a**), cell cycle (**b**), and macrophage M1 polarization (**c**). Expression of each gene is derived from RNA sequencing of monocyte-derived macrophages at baseline (**a, b**) and after 10 h of LPS+IFNgamma treatment (**c**). Hierarchical clustering was done using Euclidean distance measure. Light blue bars represent TET2delA carriers (+/-) and grey bars controls (+/+).

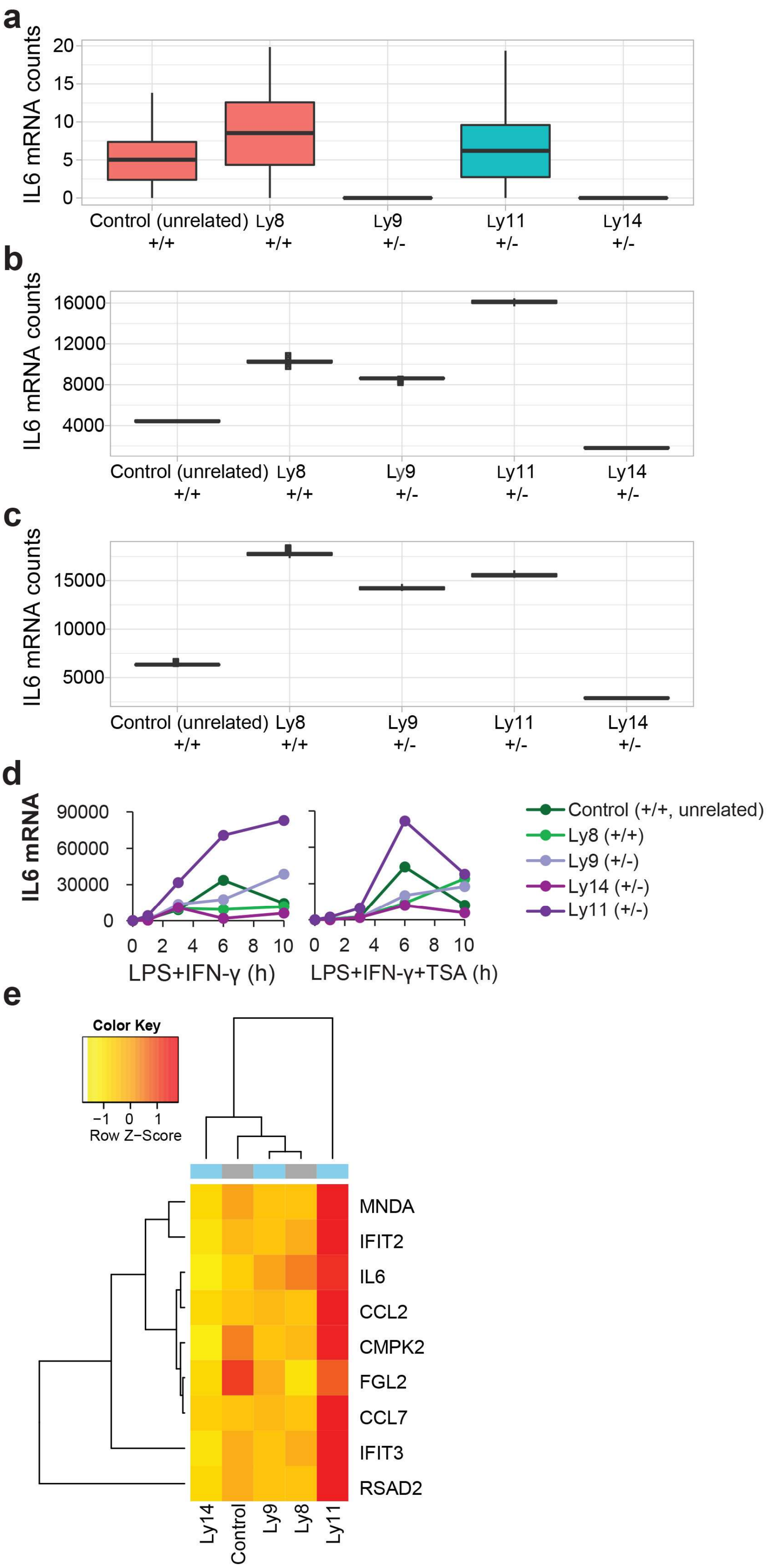

a
IL6 mRNA counts
20
15
10
5
0
Control (unrelated) +/+
Ly8 +/+
Ly9 +/-
Ly11 +/-
Ly14 +/-
b
IL6 mRNA counts
16000
12000
8000
4000
Control (unrelated) +/+
Ly8 +/+
Ly9 +/-
Ly11 +/-
Ly14 +/-
c
IL6 mRNA counts
15000
10000
5000
Control (unrelated) +/+
Ly8 +/+
Ly9 +/-
Ly11 +/-
Ly14 +/-
d
IL6 mRNA
90000
60000
30000
0
0 2 4 6 8 10
LPS+IFN-γ (h)
0 2 4 6 8 10
LPS+IFN-γ+TSA (h)
Control (+/+, unrelated)
Ly8 (+/+)
Ly9 (+/-)
Ly14 (+/-)
Ly11 (+/-)
e
Color Key
−1 0 1
Row Z−Score
MNDA
IFIT2
IL6
CCL2
CMPK2
FGL2
CCL7
IFIT3
RSAD2
Ly14
Control
Ly9
Ly8
Ly11

Supplementary Figure 21. Expression of IL6 and eight similarly TET2-HDAC2-repressed LPS-inducible target genes reported in mice studied in macrophages of *TET2* mutation carriers and controls (previous page). Expression of IL6 as measured by RNA sequencing in monocyte-derived macrophages at baseline (**a**), after 10 h of LPS+IFNgamma treatment (**b**), and after 10 h of LPS+IFNgamma treatment in the presence of trichostatin A (TSA) (**c**). IL-6 expression in macrophages was additionally analyzed by quantitative PCR in macrophages of TET2delA carriers and controls (**d**). (**e**) Expression heatmap of IL6 and eight further LPS-inducible transcripts from Zhang et al.[28] in macrophages after after 10 h of LPS+IFNgamma treatment. Hierarchical clustering was done using Euclidean distance measure. Light blue bars represent TET2delA carriers (+/-) and grey bars controls (+/+).

Supplementary Table 1. Clinical features, immunophenotypes, and outcomes of the nodular lymphocyte predominant Hodgkin lymphoma patients in the family

Patient	Gender	Age at diagnosis	Immunophenotype	Stage	Treatment	Relapse	Treatment for relapse	Current status
Ly1	F	46	CD20+, CD30-, CD15-, CD3-	IA	ABVD-radiotherapy	No	No	Remission
Ly2	M	45	CD20+, CD30-, CD15-, CD3-	IIIA	ABVD-radiotherapy	1) A suspected relapse in the spine 4 years later<br>2) T-cell rich B-cell lymphoma 7 years later	1) Radiotherapy<br>2) R-CHOEP-radiotherapy	Remission
Ly3	M	39	CD20+, CD30-, CD15-, CD3-	IIIB	MOPP/ABV-radiotherapy	Mixed-cellular Hodgkin lymphoma 2 years later	MOPP/ABVD	Deceased

ABVD, doxorubicin, bleomycin, vinblastine, decarbazine
MOPP/ABV, methotrexate, oncovin, procarbazine, prednisone/adriamycin, bleomycin, vinblastine
MOPP/ABVD, methotrexate, oncovin, procarbazine, prednisone/adriamycin, bleomycin, vinblastine, dacarbazine
R-CHOEP, rituximab, cyclophosphamide, hydroxydaunorubicin, oncovin, etoposide, prednisone

Supplementary Table 2. The list of shared likely damaging variants in three siblings with nodular lymphocyte predominant Hodgkin lymphoma; final candidate variants remaining after filtering in grey background

Gene Name	Ensembl Gene ID	Position	Variant (ref/alt)	db SNP 144	Variant Quality Score[1]	Provean Prediction	SIFT Prediction	ExAC Allele Count[2]	1kG AGM[3]	Sanger validation
DMRTA2	ENSG00000142700	1:50884778	GGCGGC/-		93	na	na	0	NO	nd
ZNF717	ENSG00000227124	3:75787349	CCCTG/TAA	rs386662583	221	na	na	0	NO	nd
ZNF717	ENSG00000227124	3:75787519	CT/AG		225	Deleterious	Damaging	0	NO	nd
TET2	ENSG00000168769	4:106194035	A/-		428	na	na	0	YES	TRUE
PCDHA4	ENSG00000204967	5:140186979	GGGCCGCGGAGG/AAGACACCGGGA		1099	na	na	0	NO	nd
PCYOX1L	ENSG00000145882	5:148743741	G/A		391	Deleterious	Damaging	0	YES	TRUE
ERVFRD-1	ENSG00000244476	6:11104214	G/A		567	na	na	0	NO	nd
HLA-A	ENSG00000206503	6:29910716	CA/GG	rs386698551	392	Deleterious	na	0	NO	nd
HLA-A	ENSG00000206503	6:29911114	GG/AG,AC	rs66488547	274	na	na	0	NO	nd
HLA-B	ENSG00000234745	6:31324641	TGG/AGC		115	Deleterious	na	0	NO	nd
HLA-DPB1	ENSG00000223865	6:33048686	GCGGGCCCA/ACGAGGCCG		386	na	na	5681	NO	nd
ABRA	ENSG00000174429	8:107781957	GTGGCTGT/-	rs751237288	926	na	na	0	YES	TRUE, but healthy mother homozygous
GPSM1	ENSG00000160360	9:139252651	G/CC		111	na	na	0	NO	nd
AL450307.1	ENSG00000189275	10:133608291	TT/GG	rs35760301	671	Deleterious	na	799	YES	nd
OR4C3	ENSG00000176547	11:48347140	TACGT/CATGC	rs386753296	214	Deleterious	na	10015	NO	nd
OR8U1	ENSG00000172199	11:56143906	CA/TG		589	Deleterious	Damaging	6	NO	nd
OR9G1	ENSG00000174914	11:56468198	ACCTG/GCTTA		87	Deleterious	Damaging	10101	NO	nd
EMP1	ENSG00000134531	12:13366642	T/C	rs201135816	537	Deleterious	Damaging	5	YES	nd
OR8S1	ENSG00000197376	12:48919659	TG/CA	rs71439450	1027	Deleterious	Damaging	7055	YES	nd
CELA1	ENSG00000139610	12:51740388	CTGGACCATATCCACTTACCATAAAGGAC/ACACCAGGAAGCG	rs386762976	904	na	na	0	YES	TRUE
KRT2	ENSG00000172867	12:53045626	T/CGCTGCCGCCTCCAAAGCC		480	na	na	0	NO	nd
DIS3	ENSG00000083520	13:73333956	T/A	rs778587104	403	na	na	1	YES	nd
AC135048.1	ENSG00000268863	16:30996871	CG/GC	rs71380413	75	na	na	91	NO	nd
CDC27	ENSG00000004897	17:45214631	G/A	rs11570544	670	Deleterious	Damaging	3086	NO	nd
ICAM5	ENSG00000105376	19:10402256	-/C		149	na	na	0	NO	nd
IER2	ENSG00000160888	19:13264425	G/T	rs760342538	364	Deleterious	Damaging	3	NO	nd

na=not available; nd=not determined

[1]Variant quality score for Ly1

[2]Total number of variant alleles in the Exome Aggregation Consortium (ExAC, version 2.0) data

[3]1 kG AGM, 1000 Genomes Project Accessibility Genome Mask, strict; YES indicates that the variant lies inside an accessible genomic region to accurate analysis

Supplementary Table 3. Samples used in methylation analyses

Individual name (identifier)	Gender	Disease status	*TET2/DNMT3A* mutation status	Year of blood DNA extraction	Age at sample collection
**Targeted bisulfite sequencing**					
Ly1	female	NLPHL	TET2delA	2007, 2009	54, 57
Ly2	male	NLPHL, TCRBCL	TET2delA	2007, 2009	52, 55
Ly8	female	healthy	wild-type	2007	32
Ly9	female	healthy	TET2delA	2007	31
Ly10	male	healthy	wild-type	2007	26
Ly11	female	healthy	TET2delA	2007	26
Ly12	male	healthy	wild-type	2016	19
Ly13	male	healthy	wild-type	2016	20
Ly14	female	healthy	TET2delA	2016	19
HLRCC_N7	female	HLRCC	wild-type	2007	32
Control1	female	healthy	nd	2007	42
Control2	male	healthy	nd	2007	28
Control3	female	healthy	nd	2007	30
Control4	female	healthy	nd	2009	44
Control5	female	healthy	nd	2009	68
Id5 (SL43QTGQOHCB)	male	TBRS	DNMT3A p.Arg736Pro	2014	8
Id6 (N3MKS6FWMIVF)	male	ID	wild-type	2014	7
Id7 (NITBUAAMODVL)	female	TBRS	DNMT3A p.Gly707Fs	2014	7
Id8 (UJRZ2ZGPP6EA)	female	ID	wild-type	2014	7
Id9 (ZQYJY6DPCSHJ)	male	Likely TBRS	DNMT3A p.Tyr735Cys	2013	17
Id10 (OBLCHPDRWBEX)	male	ID	wild-type	2013	17
Id11 (RVGOKOYNIGRV)	female	ID	DNMT3A p.Asp194Fs	2015	39
Id12 (YVENDJGVGNTC)	female	ID	wild-type	2015	39
**Whole-genome bisulfite sequencing**					
Ly8	female	healthy	wild-type	2017	42
Ly9	female	healthy	TET2delA	2017	41
Ly10	male	healthy	wild-type	2017	36
Ly11	female	healthy	TET2delA	2017	36
Ly13	male	healthy	wild-type	2017	21
Ly14	female	healthy	TET2delA	2017	20
Id1 (2VLH7VYEOG6D)	male	ID	TET2X	2014	16
Id2 (MTFSEZTNNRN4)	male	ID	wild-type	2013	16
Id3 (AR3NWS45ZYJV)	male	ID	wild-type	2016	16

NLPHL: Nodular lymphocyte predominant Hodgkin's lymphoma, TCRBCL: T-cell rich B-cell lymphoma, HLRCC: Hereditary leiomyomatosis and renal cell cancer syndrome, nd: not determined, TBRS: Tatton-Brown-Rahman syndrome, ID: intellectual disability

Supplementary Table 4. Alignment statistics of targeted bisulfite-sequenced samples

Sample	Sequence pairs in total	Alignment to top strand %	Alignment to bottom strand %	Duplicate percentage	Average depth at target regions	Percentage of target regions with depth ≥ 6	C methylated in CpG context	Average depth at targeted CpGs
Ly1 -07	51832004	16%	77%	22.0 %	50	93%	55.60%	17
Ly1 -09	38279859	34%	58%	1.5 %	19	84%	65.30%	8
Ly2 -07	42178602	18%	75%	13.5 %	43	92%	54.80%	14
Ly2 -09	58587789	34%	57%	1.6 %	29	90%	66.50%	10
Ly8 -07	54732660	18%	75%	12.6 %	57	94%	55.20%	18
Ly9 -07	34545158	10%	84%	18.0 %	42	92%	55.80%	16
Ly10 -07	67751542	11%	82%	30.8 %	67	94%	56.10%	21
Ly11 -07	46577029	10%	84%	13.2 %	61	94%	56.30%	21
Ly12 -16	22076997	4%	87%	9.8 %	38	92%	50.70%	18
Ly13 -16	22228769	11%	77%	6.3 %	33	90%	53.20%	15
Ly14 -16	22178559	16%	74%	4.5 %	29	90%	55.30%	13
HLRCC_N7 -07	55940064	15%	78%	45.2 %	39	91%	54.90%	14
Control1 -07	27509084	7%	88%	29.8 %	31	87%	54.40%	12
Control2 -07	35678908	7%	86%	34.1 %	37	89%	56.20%	14
Control3 -07	40869862	8%	86%	14.9 %	54	93%	54.20%	19
Control4 -09	43177853	31%	60%	1.5 %	25	88%	64.20%	9
Control5 -09	67064104	33%	58%	3.5 %	35	92%	63.60%	11
Id5	39563390	4%	86%	13.44%	65	94%	52.20%	27
Id6	40683265	4%	86%	15.59%	64	95%	53.60%	27
Id7	32380378	4%	85%	14.24%	52	93%	52.50%	22
Id8	42295429	4%	87%	18.57%	65	95%	53.90%	27
Id9	36171739	4%	82%	21.62%	51	93%	53.40%	22
Id10	36877881	4%	86%	12.15%	62	95%	52.60%	27
Id11	35597938	4%	86%	11.96%	59	95%	50.80%	26
Id12	34062681	4%	87%	17.75%	55	93%	54.20%	22

Supplementary Table 5. Number of differentially methylated cytosines per sample as compared to five baseline controls

Sample (*TET2* or *DNMT3A* mutation status)	Significant hyper CpGs (methylation difference >0.2)	Significant hypo CpGs (methylation difference <-0.2)	Total sites tested	Hyper counts per million tested CpGs	Hypo counts per million tested CpGs
Ly1-07 (*TET2*+/-)	793	364	3168315	250.29	114.89
Ly1-09 (*TET2*+/-)	1097	559	2718156	403.58	205.65
Ly2-07 (*TET2*+/-)	810	616	3117150	259.85	197.62
Ly2-09 (*TET2*+/-)	1036	549	2992321	346.22	183.47
Ly9-07 (*TET2*+/-)	1369	402	3088501	443.26	130.16
Ly11-07 (*TET2*+/-)	925	427	3235621	285.88	131.97
Ly14-16 (*TET2*+/-)	1036	843	3012948	343.85	279.79
Ly8-07 (*TET2*+/+)	511	793	3271178	156.21	242.42
Ly10-07 (*TET2*+/+)	556	719	3240371	171.59	221.89
Ly12-16 (*TET2*+/+)	353	1479	3148460	112.12	469.75
Ly13-16 (*TET2*+/+)	427	1175	3037046	140.60	386.89
HLRCC_N7 (*TET2*+/+)	355	933	3036415	116.91	307.27
Id5 (*DNMT3A*+/-)	373	6086	3364416	110.87	1808.93
Id6 (*DNMT3A*+/+)	2159	1125	3401695	634.68	330.72
Id7 (*DNMT3A*+/-)	358	5671	3272881	109.38	1732.72
Id8 (*DNMT3A*+/+)	813	783	3374528	240.92	232.03

Id9 (*DNMT3A*+/-)	377	4480	3202371	117.73	1398.96
Id10 (*DNMT3A*+/+)	793	827	3388987	233.99	244.03
Id11 (*DNMT3A*+/-)	491	2183	3372253	145.60	647.34
Id12 (*DNMT3A*+/+)	1143	747	3223934	354.54	231.70

Supplementary Table 6. Methylation differences between *TET2* c.4500delA mutation carriers (n=5) and controls (n=10), and permutation p-values for the lineage-specific open chromatin regions with master transcription factor binding sequence

		Bcell	CD4	CD8	CLP	CMP	GMP	HSC	MEP	Mono	Nkcell
**PU.1**	**With motif (full overlap)**	4234	1321	856	938	14124	7890	7030	5732	1306	1134
	**W/o motif (full exclusion)**	25760	24428	17808	9036	55927	27578	36254	77713	5631	26029
	**Fraction with motif**	0.14	0.05	0.05	0.09	0.20	0.22	0.16	0.07	0.19	0.04
	**Methylation difference (%)**	3.41	1.82	1.38	3.92	2.48	4.19	2.38	2.09	4.34	1.15
	**FDR (1000 permutations, two-sided p-value)**	0.013	0.664	0.867	0.646	0.000	0.033	0.000	0.169	0.871	0.867
**RUNX**	**With motif (full overlap)**	2806	3919	3372	1213	10869	4751	6442	9564	616	4376
	**W/o motif (full exclusion)**	27194	21813	15285	8774	59182	30725	36864	73876	6324	22778
	**Fraction with motif**	0.09	0.15	0.18	0.12	0.16	0.13	0.15	0.11	0.09	0.16
	**Methylation difference (%)**	2.50	2.05	2.17	2.52	1.89	3.43	1.79	1.89	5.88	1.37
	**FDR (1000 permutations, two-sided p-value)**	0.709	0.033	0.360	0.867	0.050	0.867	0.000	0.135	0.576	0.094
**CEBPB**	**With motif (full overlap)**	1020	944	645	273	4535	3782	1701	2965	1099	1089
	**W/o motif (full exclusion)**	28985	24804	18028	9715	65536	31711	41620	80504	5841	26087
	**Fraction with motif**	0.03	0.04	0.03	0.03	0.06	0.11	0.04	0.04	0.16	0.04
	**Methylation difference (%)**	5.83	1.60	2.87	3.99	1.71	2.65	1.79	2.24	4.77	0.57
	**FDR (1000 permutations, two-sided p-value)**	0.000	0.867	0.240	0.867	0.533	0.395	0.135	0.173	0.937	0.466
**GATA**	**With motif (full overlap)**	1668	1911	1261	524	13448	1965	4253	16173	335	1587
	**W/o motif (full exclusion)**	28353	23840	17410	9463	56617	33542	39070	67280	6609	25591
	**Fraction with motif**	0.06	0.07	0.07	0.05	0.19	0.06	0.10	0.19	0.05	0.06
	**Methylation difference (%)**	0.73	1.66	1.96	2.81	1.89	2.48	1.81	1.60	5.46	1.13
	**FDR (1000 permutations, two-sided p-value)**	0.094	0.698	0.867	0.942	0.000	0.576	0.013	0.432	0.867	0.867
**PAX5**	**With motif (full overlap)**	2217	836	606	1662	2090	1088	1346	2690	247	1060
	**W/o motif (full exclusion)**	27758	24904	18055	8314	67946	34386	41967	80718	6695	26096
	**Fraction with motif**	0.07	0.03	0.03	0.17	0.03	0.03	0.03	0.03	0.04	0.04
	**Methylation difference (%)**	1.90	1.10	1.62	0.34	1.45	3.10	0.76	1.30	4.86	0.87
	**FDR (1000 permutations, two-sided p-value)**	0.904	0.867	0.937	0.000	0.937	0.942	0.276	0.937	0.937	0.937
**TBX21**	**With motif (full overlap)**	4144	3342	3192	1371	8715	4549	5561	10625	930	5118

**W/o motif (full exclusion)**	25896	22434	15495	8624	61430	30983	37810	72907	6022	22083
**Fraction with motif**	0.14	0.13	0.17	0.14	0.12	0.13	0.13	0.13	0.13	0.19
**Methylation difference (%)**	2.20	1.45	2.19	2.69	1.46	3.16	1.45	1.52	3.61	1.63
**FDR (1000 permutations, two-sided p-value)**	0.946	0.937	0.343	0.937	0.937	0.937	0.360	0.867	0.576	0.000

CLP: common lymphoid progenitor; CMP: common myeloid progenitor; GMP: granulocyte/macrophage progenitor; HSC: hematopoietic stem cell; MEP: megakaryocyte/erythroid progenitor; Mono: monocyte

Supplementary Table 7. Leukocyte counts of *TET2* c.4500delA (TET2delA) and c.1471C>T (TET2X) carriers

Arrows indicate deviation from the reference values.

Patient	Control Range	Ly1* (TET2delA)	Ly2* (TET2delA)	Ly9 (TET2delA)	Ly11 (TET2delA)	Ly14 (TET2delA)	Id1 (TET2X)
**Leukocytes**	3400-8200	5400	5400	9400 ↑	8200	7800	5200
**Lymphocytes**	1300-3600	2100	2100	4200 ↑	1900	3000	2700
**Neutrophils**	1500-6700	2600	2600	4300	5100	3900	2100
**Monocytes**	200-800	600	600	900 ↑	1000 ↑	600	500
**Basophils**	0-100	50	20	20	30	80	20
**Eosinophils**	30-440	60	80	60	160	100	30
**B-cells (CD19+)**	100-500	340	160	760 ↑	270	1300 ↑	410
**CD3+CD4+**	300-1400	530	920	1080	990	1500 ↑	1140
**CD3+CD8+**	200-1200	470	870	750	190 ↓	660	800
**NK-cells ($CD3^{-}CD16^{+}56^{+}$)**	90-600	290	160	290	250	240	240

*mutation carriers, who have been treated for nodular lymphocyte predominant Hodgkin lymphoma

Supplementary Table 8. B- and T-cell differentials of *TET2* c.4500delA (TET2delA) and c.1471C>T (TET2X) carriers
Arrows indicate deviation from the reference values.

Patient	Cell type	Control Range (%)	Ly1* (TET2delA)	Ly2* (TET2delA)	Ly9 (TET2delA)	Ly11 (TET2delA)	Ly14 (TET2delA)	Id1 (TET2delX)
**$CD19^+$ B-cells**		5-22	14	8	14	12	13	15
**Transitional**	$CD38^{hi}IgM^{hi}$	0.6-3.5	2.7	4.4 ↑	4.3 ↑	1.8	0.4 ↓	2.7
**Naive**	$CD27^-IgD^+$	43.2-82.4	85.4 ↑	86.9 ↑	63.4	82.0	62.6	72.0
**Memory**	$CD27^+$	15.0-45.0	11.6 ↓	8.6 ↓	32.8	15.0	33.2	25.7
**Marginal zone-like**	$CD27^+IgD^+IgM^+$	7.2-30.8	2.5 ↓	7.0 ↓	21.7	7.2	15.7	11.9
**Switched memory**	$CD27^+IgD^-IgM^-$	6.5-29.2	3.4 ↓	0.5 ↓	8.6	6.4 ↓	13.0	9.2
**Plasmablasts**	$CD38^{++}IgM^-$	—	< 0.2	0.2	<0.2	0.3	1.0	<0.2
**Activated**	$CD38^{low}CD21^{low}$	0.6-3.5	8.4 ↑	8.2 ↑	6.4 ↑	5.0 ↑	9.6 ↑	4.3 ↑
**CD3+ T-cells**		65.9–87.6	68.0	84.0	75.0	71.0	75.0	76.0
**Double-negative**	CD4-CD8-TCRab+	0.3-3.3	1.4	1.3	1.9	0.6	nd	1.0
**$T_{\gamma\delta}$**	TCRgd+	1.9-11.7	3.0	5.7	7.7	2.3	nd	5.6
**CD3+CD4+ T-cells**		35.6–56.0	37.2	43.3	55.0	85.0 ↑	65.0 ↑	38.6
**RTE**	$CD45RA^+CD62L^+CD31^+$	14.4-38.3	10.0 ↓	5.0 ↓	17.9	30.2	47.5 ↑	47.8 ↑
**Naïve**	$CCR7^+CD45RA^+$	20.5-54.8	26.2	21.5	30.0	56.2 ↑	63.7 ↑	59.8 ↑
**TCM**	$CCR7^+CD45RA^-$	8.4-32.8	31.3	49.8 ↑	40.1 ↑	35.2 ↑	25.4	29.3
**TEM**	$CCR7^-CD45RA^-$	19.9-52.4	40.6	18.7 ↓	16.1 ↓	7.8 ↓	9.4 ↓	15.8 ↓
**TEMRA**	$CCR7^-CD45RA^+$	1.4-17.0	1.9	10.0	13.7	0.7 ↓	1.4	1.0 ↓
**Activated**	$HLADR^+CD38^-$	2.4-9.6	7.1	6.0	6.0	3.8	1.8 ↓	4.0
	$HLADR^-CD38^+$	40.4-72.9	45.0	50.7	50.5	59.8	62.5	70.1
	$HLADR^+CD38^+$	0.9-4.6	6.0	22.6 ↑	8.5 ↑	3.8	5.7 ↑	2.1
**Treg**	$CD25^{hi}CD127^{lo}$	2.8-6.4	3.2	2.2 ↓	2.6 ↓	6.0	4.6	4.9
**CD3+CD8+ T-cells**		13.1–34.5	33.0	41.0	38.0	16.4	28.7	15.3
**Naive**	$CCR7^+CD45RA^+$	18.8-71.0	7.1 ↓	3.8 ↓	27.2	53.0	62.4	65.8
**TCM**	$CCR7^+CD45RA^-$	1.2-7.3	0.9 ↓	2.4	7.5 ↑	4.5	2.9	2.0
**TEM**	$CCR7^-CD45RA^-$	14.6-63	7.4 ↓	23.7	9.7 ↓	4.2 ↓	7.4 ↓	6.9 ↓
**TEMRA**	$CCR7^-CD45RA^+$	4.5-33.7	84.6 ↑	70.0 ↑	55.6 ↑	38.3 ↑	27.3	25.3

**Activated**	HLADR^{+}CD38^{-}	3.8-32.4	13.5	9.9	15.7	6.2	8.7	4.0
	HLADR^{-}CD38^{+}	30.3-78.5	24.9	38.9	32.8	53.1	54.5	64.8
	HLADR^{+}CD38^{+}	1.4-21.4	7.3	30.6 ↑	31.3↑	9.5	12.8	3.5

*mutation carriers, who have been treated for nodular lymphocyte predominant Hodgkin lymphoma

Supplementary Table 9. Twenty most upregulated and downregulated genes between cancer-free *TET2* c.4500delA (TET2delA) and age-matched wild-type individuals (wt) in NK cells and CD8+ T cells

Ensembl ID	Gene Name	delA Average	wt Average	Log2 Fold Change	P-Value
ENSG00000121966	CXCR4	1.34	0.32	2.06	0.000
ENSG00000157514	TSC22D3	1.48	0.67	1.14	0.002
ENSG00000133639	BTG1	4.31	2.56	0.75	0.374
ENSG00000152518	ZFP36L2	2.44	1.56	0.64	0.934
ENSG00000160310	PRMT2	1.4	0.92	0.6	1.000
ENSG00000115523	GNLY	27.26	18.25	0.58	1.000
ENSG00000198034	RPS4X	15.36	10.6	0.53	1.000
ENSG00000019582	CD74	1.97	1.43	0.46	1.000
ENSG00000223865	HLA-DPB1	1.18	0.91	0.38	1.000
ENSG00000115738	ID2	2.94	2.3	0.35	1.000
ENSG00000100911	PSME2	1.19	0.95	0.33	1.000
ENSG00000104894	CD37	1.39	1.16	0.26	1.000
ENSG00000198851	CD3E	1.22	1.02	0.26	1.000
ENSG00000117289	TXNIP	5.58	4.68	0.25	1.000
ENSG00000116824	CD2	1.77	1.49	0.25	1.000
ENSG00000140988	RPS2	16.86	14.25	0.24	1.000
ENSG00000181163	NPM1	2.29	1.94	0.24	1.000
ENSG00000105372	RPS19	19.6	16.75	0.23	1.000
ENSG00000135046	ANXA1	1.87	1.6	0.22	1.000
ENSG00000169442	CD52	5.42	4.66	0.22	1.000
ENSG00000051523	CYBA	4.23	5.21	-0.3	1.000
ENSG00000127314	RAP1B	1.61	1.99	-0.3	1.000
ENSG00000155368	DBI	1.27	1.58	-0.31	1.000
ENSG00000099622	CIRBP	1.22	1.52	-0.32	1.000
ENSG00000026025	VIM	1.34	1.67	-0.32	1.000
ENSG00000172531	PPP1CA	1.06	1.33	-0.32	1.000
ENSG00000011600	TYROBP	2.92	3.73	-0.35	1.000
ENSG00000141002	TCF25	0.93	1.19	-0.35	1.000
ENSG00000164924	YWHAZ	1.02	1.3	-0.35	1.000
ENSG00000197971	MBP	0.81	1.03	-0.35	1.000
ENSG00000198786	MT-ND5	2.88	3.72	-0.37	1.000
ENSG00000100097	LGALS1	1.26	1.68	-0.41	1.000
ENSG00000115956	PLEK	0.96	1.28	-0.42	1.000
ENSG00000198821	CD247	1.31	1.82	-0.47	1.000
ENSG00000099795	NDUFB7	0.7	1.07	-0.62	1.000
ENSG00000159674	SPON2	1.01	1.57	-0.64	1.000
ENSG00000185885	IFITM1	0.89	1.6	-0.85	0.271
ENSG00000197728	RPS26	2.72	5.53	-1.02	0.024
ENSG00000107317	PTGDS	0.1	1.29	-3.7	0.000
ENSG00000129824	RPS4Y1	0	1.39	-9.71	0.000

Supplementary Table 10. Twenty most upregulated and downregulated genes between cancer-free *TET2* c.4500delA (TET2delA) and age-matched wild-type individuals (wt) in CD4+ T cells

Ensembl ID	Gene Name	delA Average	wt Average	Log2 Fold Change	P-Value
ENSG00000121966	CXCR4	1.91	0.88	1.12	0.013
ENSG00000113088	GZMK	1.44	0.75	0.94	0.605
ENSG00000229807	XIST	1.06	0.56	0.91	0.397
ENSG00000115523	GNLY	1.05	0.61	0.77	1.000
ENSG00000157514	TSC22D3	1.56	0.91	0.77	1.000
ENSG00000126353	CCR7	1.15	0.74	0.64	1.000
ENSG00000145649	GZMA	1.02	0.69	0.57	1.000
ENSG00000163220	S100A9	1.09	0.74	0.56	1.000
ENSG00000133639	BTG1	5.82	4.12	0.5	1.000
ENSG00000105374	NKG7	1.77	1.25	0.49	1.000
ENSG00000161570	CCL5	3.06	2.21	0.47	1.000
ENSG00000111796	KLRB1	2.63	1.93	0.45	1.000
ENSG00000152518	ZFP36L2	1.97	1.48	0.41	1.000
ENSG00000266402	SNORA76	1.78	1.35	0.4	1.000
ENSG00000019582	CD74	1.52	1.16	0.39	1.000
ENSG00000204525	HLA-C	8.71	6.8	0.36	1.000
ENSG00000196329	GIMAP5	1.2	0.97	0.31	1.000
ENSG00000188404	SELL	1.36	1.1	0.31	1.000
ENSG00000213719	CLIC1	1.2	0.98	0.3	1.000
ENSG00000144746	ARL6IP5	1.11	0.93	0.25	1.000
ENSG00000125691	RPL23	9.42	10.32	-0.13	1.000
ENSG00000171223	JUNB	1.14	1.25	-0.13	1.000
ENSG00000063046	EIF4B	1.21	1.33	-0.14	1.000
ENSG00000169442	CD52	7.29	8.03	-0.14	1.000
ENSG00000220205	VAMP2	0.92	1.02	-0.14	1.000
ENSG00000115268	RPS15	20.45	22.66	-0.15	1.000
ENSG00000185787	MORF4L1	0.9	1	-0.16	1.000
ENSG00000100650	SRSF5	1.47	1.64	-0.16	1.000
ENSG00000198888	MT-ND1	11.18	12.55	-0.17	1.000
ENSG00000103363	TCEB2	1	1.13	-0.17	1.000
ENSG00000099622	CIRBP	1.32	1.5	-0.18	1.000
ENSG00000183172	SMDT1	1.06	1.23	-0.21	1.000
ENSG00000026025	VIM	3.04	3.67	-0.27	1.000
ENSG00000198763	MT-ND2	17.96	21.79	-0.28	1.000
ENSG00000142546	NOSIP	1.4	1.75	-0.32	1.000
ENSG00000231500	RPS18	33.1	45.84	-0.47	1.000
ENSG00000198786	MT-ND5	2.48	3.55	-0.52	1.000
ENSG00000269893	SNHG8	1.23	1.78	-0.53	1.000
ENSG00000197728	RPS26	5.57	9.17	-0.72	1.000
ENSG00000129824	RPS4Y1	0	1.99	-10.82	0.000

Supplementary Table 11. Twenty most upregulated and downregulated genes between cancer-free *TET2* c.4500delA (TET2delA) and age-matched wild-type individuals (wt) in B cells

Ensembl ID	Gene Name	delA Average	wt Average	Log2 Fold Change	P-Value
ENSG00000157514	TSC22D3	1.23	0.89	0.46	1.000
ENSG00000204525	HLA-C	6	4.62	0.38	1.000
ENSG00000198034	RPS4X	21.21	16.56	0.36	1.000
ENSG00000170476	MZB1	1.59	1.29	0.3	1.000
ENSG00000125534	PPDPF	1.65	1.36	0.28	1.000
ENSG00000121966	CXCR4	2.31	1.94	0.25	1.000
ENSG00000125743	SNRPD2	1.44	1.21	0.25	1.000
ENSG00000165502	RPL36AL	2.94	2.5	0.23	1.000
ENSG00000147677	EIF3H	1.45	1.25	0.22	1.000
ENSG00000133639	BTG1	4.73	4.07	0.22	1.000
ENSG00000234745	HLA-B	10.08	8.75	0.2	1.000
ENSG00000088986	DYNLL1	1.03	0.9	0.2	1.000
ENSG00000127540	UQCR11	1.24	1.09	0.18	1.000
ENSG00000213988	ZNF90	1.17	1.04	0.18	1.000
ENSG00000152082	MZT2B	1.01	0.89	0.18	1.000
ENSG00000178982	EIF3K	1.47	1.3	0.18	1.000
ENSG00000120742	SERP1	1.34	1.19	0.17	1.000
ENSG00000106153	CHCHD2	1.6	1.42	0.17	1.000
ENSG00000124172	ATP5E	4.57	4.07	0.16	1.000
ENSG00000070756	PABPC1	4.78	4.28	0.16	1.000
ENSG00000212907	MT-ND4L	1.21	1.38	-0.19	1.000
ENSG00000115268	RPS15	15.15	17.29	-0.19	1.000
ENSG00000152795	HNRNPDL	1.04	1.2	-0.2	1.000
ENSG00000026025	VIM	1.61	1.86	-0.21	1.000
ENSG00000128218	VPREB3	0.91	1.06	-0.21	1.000
ENSG00000108654	DDX5	2.97	3.47	-0.23	1.000
ENSG00000198727	MT-CYB	13.28	15.78	-0.25	1.000
ENSG00000198763	MT-ND2	14.72	17.53	-0.25	1.000
ENSG00000198888	MT-ND1	10.67	12.72	-0.25	1.000
ENSG00000175567	UCP2	1.07	1.28	-0.26	1.000
ENSG00000237541	HLA-DQA2	2.09	2.51	-0.27	1.000
ENSG00000132465	IGJ	8.51	10.6	-0.32	1.000
ENSG00000254709	IGLL5	9.74	12.2	-0.33	1.000
ENSG00000166794	PPIB	1.06	1.32	-0.33	1.000
ENSG00000090382	LYZ	0.83	1.06	-0.35	1.000
ENSG00000100721	TCL1A	1.78	2.32	-0.39	1.000
ENSG00000198786	MT-ND5	2.24	3.48	-0.64	1.000
ENSG00000253701	AL928768.3	0.64	1.03	-0.68	1.000
ENSG00000197728	RPS26	2.53	4.89	-0.95	0.315
ENSG00000129824	RPS4Y1	0	1.6	-9.7	0.000

Supplementary Table 12. Twenty most upregulated and downregulated genes between cancer-free *TET2* c.4500delA (TET2delA) and age-matched wild-type individuals (wt) in monocytes

Ensembl ID	Gene Name	delA Average	wt Average	Log2 Fold Change	P-Value
ENSG00000229807	XIST	1.52	0.47	1.69	0.000
ENSG00000157514	TSC22D3	1.18	0.55	1.1	0.004
ENSG00000126709	IFI6	1.28	0.66	0.96	0.085
ENSG00000213145	CRIP1	3.23	1.68	0.94	0.063
ENSG00000187608	ISG15	1.02	0.59	0.78	1.000
ENSG00000125148	MT2A	1.04	0.61	0.77	0.635
ENSG00000221869	CEBPD	2.29	1.37	0.74	0.468
ENSG00000143384	MCL1	1.88	1.15	0.71	0.634
ENSG00000171051	FPR1	1.14	0.74	0.61	1.000
ENSG00000197766	CFD	2.8	1.88	0.58	1.000
ENSG00000204525	HLA-C	7.1	5.1	0.48	1.000
ENSG00000257764	RP11-1143G9.4	6.32	4.64	0.45	1.000
ENSG00000158710	TAGLN2	1.41	1.04	0.44	1.000
ENSG00000237541	HLA-DQA2	1.21	0.9	0.43	1.000
ENSG00000135046	ANXA1	3.56	2.65	0.42	1.000
ENSG00000234745	HLA-B	14.54	10.84	0.42	1.000
ENSG00000160255	ITGB2	2.48	1.85	0.42	1.000
ENSG00000170458	CD14	3.16	2.36	0.42	1.000
ENSG00000116741	RGS2	1.33	1	0.41	1.000
ENSG00000266402	SNORA76	1.4	1.07	0.38	1.000
ENSG00000093072	CECR1	0.88	1.02	-0.21	1.000
ENSG00000081189	MEF2C	1.06	1.22	-0.21	1.000
ENSG00000169442	CD52	2.36	2.77	-0.23	1.000
ENSG00000108654	DDX5	2.39	2.8	-0.23	1.000
ENSG00000121316	PLBD1	0.92	1.08	-0.23	1.000
ENSG00000241399	CD302	0.86	1.01	-0.23	1.000
ENSG00000198786	MT-ND5	3.53	4.25	-0.27	1.000
ENSG00000198830	HMGN2	1.09	1.32	-0.27	1.000
ENSG00000112799	LY86	0.87	1.06	-0.28	1.000
ENSG00000042493	CAPG	0.99	1.21	-0.28	1.000
ENSG00000153113	CAST	1.01	1.23	-0.29	1.000
ENSG00000187109	NAP1L1	2.03	2.49	-0.29	1.000
ENSG00000057608	GDI2	0.86	1.06	-0.29	1.000
ENSG00000005022	SLC25A5	1.17	1.45	-0.3	1.000
ENSG00000028277	POU2F2	0.88	1.12	-0.35	1.000
ENSG00000231500	RPS18	15.61	20.25	-0.38	1.000
ENSG00000143669	LYST	0.82	1.11	-0.43	1.000
ENSG00000197728	RPS26	3.54	4.86	-0.46	1.000
ENSG00000110203	FOLR3	0.3	1.3	-2.12	0.000
ENSG00000129824	RPS4Y1	0	1.55	-9.39	0.000

Supplementary Table 13. PCR and Sanger sequencing primers used in the study

Target gene	Primer	Sequence 5' -> 3'
TET2	TET2_ex10_FFPE_2_F	GAGAAAAAACGGAGTGGTGC
	TET2_ex10_FFPE_2_R	ACTTACAAGTTGATGGGGGC
	TET2_ex10_F-1	AGACTTGCCGACAAAGGAAA
	TET2_ex10_R-1	GGGGGCAAAACCAAAATAAT
	TET2_3-4_F	ACTCACCCATCGCATACCTC
	TET2_3-4_R	AGATAGTGCTGTGTTGGGGG
	TET2_3-5_F	TTCCACAGGTTCCTCAGCTT
	TET2_3-5_R	GAGAAGTGCACCTGGTGTGA
PCYOX1L	PCYOX1L_ex3_F	TTCATGCTGGAGGAGACTGA
	PCYOX1L_ex3_R	TACAGGCAAGCTGGTTCCTC
CELA1	CELA1_ex1_1_F	CCCCCTCCCTATAGACCTCA
	CELA1_ex1_1_R	GCCAGTTGGCAAGAAGACAG
ABRA	ABRA_ex1_F	CTCCTGCTCCATCACTCTCC
	ABRA_ex1_R	AAAACGGTGGTCAGCAAGAC

F, forward primer; R, reverse primer.

Supplementary Table 14. Single-cell capture and sequencing statistics

Individual name (TET2delA status)	Approximate time in hours from sampling to capture	Average cell viability	Estimated cell capture (based on cDNA yield)	Estimated number of cells (based on cellranger count)	Sequencing saturation
Ly1 (+/-)	24	69.53 %	1700	2444	92.80%
Ly2 (+/-)	24	66.23 %	1700	2341	89.30%
Ly8 (+/+)	5	56.45 %	1500	1107	94.10%
Ly9 (+/-)	5	55.15 %	1500	1294	93.80%
Ly10 (+/+)	5	60.00 %	1500	1165	94.30%
Ly11 (+/-)	6	79.43 %	1000	1076	96.40%
Ly13 (+/+)	5	64.10 %	1500	1067	94.50%
Ly14 (+/-)	5	73.30 %	1500	955	94.10%

Supplementary Table 15. Sequencing statistics of deep exome sequenced samples at capture target regions

Sample	Average coverage	Average mapping quality
Ly1 -07	262.92	46.11
Ly1 -09	250.79	45.82
Ly1 -17	221.55	45.45
Ly2 -07	260.81	45.87
Ly2 -09	252.52	45.81
Ly2 -17	227.68	45.51
Ly8 -07	258.22	45.42
Ly8 -17	233.64	45.53
Ly9 -07	221.15	45.34
Ly9 -17	216.89	45.04
Ly10 -07	256.63	45.8
Ly11 -07	278.56	46.23
Ly11 -17	218.61	45.62
Ly13 -17	223.7	45.41
Ly14 -17	219.38	45.09

Supplementary Table 16. Sequences of primers for quantitative RT-PCR and siRNAs against TET2 used in macrophage cell culture experiments.

Target gene	Primer type	Primer name	Sequence 5' -> 3'
apoptosis-associated speck-like protein containing a CARD	qPCR	ASC_F	TTGGACCTCACCGACAAGC
	qPCR	ASC_R	ATGTCGCGCAGCACGTTA
caspase-1	qPCR	CASP1_F	ATCCCACAATGGGCTCTGTTT
	qPCR	CASP1_R	CTCTTTCAGTGGTGGGCATCT
interleukin-1β	qPCR	IL1B_F	TGGCAATGAGGATGACTTGT
	qPCR	IL1B_R	GGAAAGAAGGTGCTCAGGTC
interleukin-18	qPCR	IL18_F	TCAACTCTCTCCTGTGAGAACAAA
	qPCR	IL18_R	GTCCTGGGACACTTCTCTGAAA
interleukin-6	qPCR	IL6_F	AGGAGACTTGCCTGGTGAAA
	qPCR	IL6_R	GAGGTGCCCATGCTACATTT
interleukin-8	qPCR	CXCL8_F	TCTGCAGCTCTGTGTGAAGG
	qPCR	CXCL8_R	ACTTCTCCACAACCCTCTGC
NLR family, pyrin domain containing 3	qPCR	NLRP3_F	CAACTGCAACCTCACGTCAC
	qPCR	NLRP3_R	ACGGTCAGCTCAGGCTTTTC
ribosomal protein lateral stalk subunit P0	qPCR	RPLP0_F	GAAATCCTGAGTGATGTGCAGC
	qPCR	RPLP0_R	TCGAACACCTGCTGGATGAC
tet methylcytosine dioxygenase 1	qPCR	TET1_242_F	AAGAGGAAGTCTGTTCATCCAGTT
	qPCR	TET1_242_R	GATTTCCCTGACAGCAGCAACA
tet methylcytosine dioxygenase 2	qPCR	TET2_72_F	TAGAGGGCAGCCTTGTGGAT
	qPCR	TET2_72_R	TGCCCTCAACATGGTTGGTT
	siRNA	Hs_TET2_1	CCCAGAGTCCTAATCCATCTA
	siRNA	Hs_TET2_2	AAGCTAGCGTCTGGTGAAGAA
	siRNA	Hs_TET2_3	CAAGGCAGTGCTAATGCCTAA
	siRNA	Hs_ KIAA1546_6	AAGCGAGTTCGAGACTCATAA
tet methylcytosine dioxygenase 3	qPCR	TET3_98_F	GAGATGCGGCCTCAACGAT
	qPCR	TET3_98_R	ACATGCTCCAGGAACAACCAA

F, forward primer; R, reverse primer.

Supplementary Table 17. Sequencing statistics of chromatin immunoprecipitation followed by high-throughput sequencing from lymphoblastoid cells

Individual (TET2delA status)	Sequencing type	Total nonredundant reads	Nonredundant reads percentage	Nonredundant reads in peaks[1]	FRiP[2] nonredundant reads
Ly8 (+/+)	ChIP	39920482	99.1%	22080052	0.5531
Ly9 (+/-)	ChIP	37385215	99.0%	22135813	0.5921
Ly10 (+/+)	ChIP	37085589	99.0%	21242564	0.5728
Ly11 (+/-)	ChIP	34360454	99.1%	21660297	0.6304
Ly14 (+/-)	ChIP	41407070	99.0%	25484189	0.6155
Ly8 (+/+)	Input	35717106	95.2%	na	na
Ly9 (+/-)	Input	34601573	93.9%	na	na
Ly10 (+/+)	Input	33633276	93.3%	na	na
Ly11 (+/-)	Input	32901960	92.2%	na	na
Ly14 (+/-)	Input	37854328	92.9%	na	na

[1] Peaks called with macs2 (callpeak --broad -q 0.01).

[2] FRiP, fraction of reads in peaks.

Supplementary References

1 Zhang, X. *et al.* DNMT3A and TET2 compete and cooperate to repress lineage-specific transcription factors in hematopoietic stem cells. *Nat Genet* **48**, 1014-1023, doi:10.1038/ng.3610 (2016).

2 Ley, T. J. *et al.* Genomic and epigenomic landscapes of adult de novo acute myeloid leukemia. *N Engl J Med* **368**, 2059-2074, doi:10.1056/NEJMoa1301689 (2013).
