# Supplementary material for: Impact of constitutional TET2 haploinsufficiency on molecular and clinical phenotype in humans
Source: Nat Commun. 2019 Mar 19;10:1252. doi: 10.1038/s41467-019-09198-7 (PMC6424975; doi:10.1038/s41467-019-09198-7)
Supplement: Supplementary file 3 — Description of Additional Supplementary Files [file 41467_2019_9198_MOESM3_ESM.pdf]

Description of Additional Supplementary Information

File Name: Supplementary Data 1
Description: Differentially methylated regions (DMRs) between TET2delA carriers and wild-type samples located within Ensembl v74 transcripts including 5kb upstream from the transcription start site (TSS)

File Name: Supplementary Data 2
Description: Differentially methylated regions (DMRs) between two NLPHL cases and three cancer-free TET2delA carriers located within Ensembl v74 transcripts including 5kb upstream from the transcription start site (TSS)

File Name: Supplementary Data 3
Description: Significantly dysregulated regulons in cancer-free TET2delA carriers as compared to age-matched non-carriers across all cells

File Name: Supplementary Data 4
Description: Transcription factors (TFs) and their co-expressed target genes in significantly dysregulated regulons

File Name: Supplementary Data 5
Description: Differentially expressed genes in monocyte-derived macrophages between TET2delA mutation carriers and controls after 10 h treatment with LPS+interferon-gamma

File Name: Supplementary Data 6
Description: Differentially expressed genes in monocyte-derived macrophages between TET2delA mutation carriers and controls at baseline

File Name: Supplementary Data 7
Description: Deaths by underlying cause of death (54-group short list) in the whole population of Finland in 2017
